# NEDD4L contributes to ferroptosis and cell growth inhibition in esophageal squamous cell carcinoma by facilitating xCT ubiquitination

**DOI:** 10.1038/s41420-024-02243-5

**Published:** 2024-11-18

**Authors:** Zhen Chen, Weilong Wang, Jinghan Hou, Can Gao, Meili Song, Zijun Zhao, Ruirui Guan, Jingsheng Chen, Huicheng Wu, Siti Razila Abdul Razak, Tao Han, Junbo Zhang, Lidong Wang, Nor Hazwani Ahmad, Xiumin Li

**Affiliations:** 1 Department of Gastroenterology, the First Affiliated Hospital of Xinxiang Medical University, Henan Key Laboratory of Tumor Molecular Therapy Medicine, Xinxiang, 453003 Henan Province PR China; 2 Department of Biomedical Science, Advanced Medical and Dental Institute, Universiti Sains Malaysia, Bertam 13200 Kepala Batas, Bertam, Pulau Pinang Malaysia; 3 Institute of Psychiatry and Neuroscience, Xinxiang Medical University, Xinxiang, 453000 Henan Province PR China; 4 Xinxiang Key Laboratory for Molecular Therapy of Cancer, Xinxiang Medical University, Xinxiang, 453003 Henan Province PR China; 5 Institutes of Health Central Plains, Xinxiang Medical University, Xinxiang, 453003 Henan Province PR China; 6 Department of Surgery, the Third Affiliated Hospital of Xinxiang Medical University, Xinxiang, 453003 Henan Province PR China; 7 State Key Laboratory of Esophageal Cancer Prevention & Treatment and Henan Key Laboratory for Esophageal Cancer Research of The First Affiliated Hospital, Zhengzhou University, Zhengzhou, 450052 Henan Province PR China

**Keywords:** Cancer, Cancer genetics, Cancer, Post-translational modifications, Oncogenes

## Abstract

The oncogene xCT plays an indispensable role in tumor growth by protecting cancer cells from oxidative stress and ferroptosis. Emerging evidence indicated xCT function is tightly controlled by posttranslational modifications, especially ubiquitination. However, it still remains unclear what specific regulatory mechanism of xCT by ubiquitin ligases in human cancers. Here, we reported that NEDD4L, an E3 ubiquitin ligases, inhibited esophageal squamous cell carcinoma (ESCC) tumor growth and facilitated ferroptosis by ubiquitination of xCT. NEDD4L expression was declined in ESCC and was associated with tumor invasion, lymph node metastasis and distant metastasis. Silencing NEDD4L triggered ESCC tumor growth. Meanwhile, knock down of NEDD4L prevented the accumulation of ROS, elevated the level of GSH, reduced the content of MDA in ESCC cells, thereby inhibiting ferroptosis. Mechanistically, NEDD4L directly bound to the ∆CT domain of xCT through its WW and HECT domain. More importantly, NEDD4L promoted xCT degradation by facilitating its polyubiquitination in ESCC cells. Collectively, these findings suggest that NEDD4L is crucial in governing the stability of xCT and mediating ferroptosis in ESCC.

## Introduction

Esophageal cancer ranks as the seventh most prevalent cancer in terms of incidence and mortality in the world [1]. The most common type of esophageal cancer is esophageal squamous cell carcinoma (ESCC), which accounts for more than 90% of all cases [2]. Despite notable advancements in the diagnosis and treatment of ESCC, the overall burden of ESCC has continued to rise over time, and the five-year survival rate remains less than 15%, largely attributed to its high propensity for invasion and metastasis [3]. Hence, it is imperative to characterize the potential targets or molecular mechanisms engaged in ESCC to identify more effective and novel therapeutic strategies.

In 2012, ferroptosis was first introduced by Brent R. Stockwell as a form of cell death that relies on iron and is triggered by accumulation of reactive oxygen species (ROS) [4–6]. To date, ferroptosis has been widely studied in numerous cancer types, revealing its capacity to hinder tumor progression through post-translational modifications (PTMs) [7, 8], especially ubiquitination [9, 10]. xCT (also known as solute carrier family 7 member 11, SLC7A11), is a core gene of ferroptosis that encodes a protein involved in the transport of amino acids across cell membranes [11, 12]. It is responsible for the transport of cystine, a crucial amino acid, into cells. By promoting the production of glutathione, xCT contributes to maintain cellular redox balance and protects against ferroptosis [13]. Previous study uncovered xCT could regulate ferroptosis through the ubiquitination pathway. For instance, the suppressor of cytokine signaling 2 (SOCS2) was identified as a biomarker that predicted the sensitivity of hepatocellular carcinoma (HCC) to radiation therapy by enhancing the ubiquitin-mediated degradation of xCT and facilitating ferroptosis [14]. Recent evidence showed that the 3-hydroxy-3-methylglutaryl reductase degradation (HRD1) inhibited the development of tumors and triggered ferroptosis by promoting the degradation of xCT in ovarian cancer [15]. However, no investigation has been carried out to clarify the relationship between xCT and ubiquitination in ESCC. The search for the potential ubiquitin ligases is still ongoing and the underlying mechanisms involved in the ubiquitination and xCT in human cancers remains largely unknown.

NEDD4L (neural precursor cell expressed, developmentally downregulated 4-like), is a member of the NEDD4 family of E3 ubiquitin ligases, which play vital role in the regulation of protein degradation and cellular processes [16, 17]. The NEDD4L protein contains C2 domain, 3-4 WW domains, and HECT domain [18, 19]. These domains enable the protein to bind to specific target proteins and transfer ubiquitin molecules onto them, leading to their degradation through the proteasome pathway. Notably, NEDD4L is the E3 ubiquitin ligase most closely associated with xCT according to the prediction of Ubi Browser database (Supplementary Fig. S1 and Supplementary Table S2).

Here, we demonstrated NEDD4L functioned as a promoter for ferroptosis pathway through suppressing xCT. NEDD4L expression was low and served as a good prognosis marker in clinical samples, as well as negatively regulated xCT expression and blocked tumor growth in ESCC. Mechanistically, depletion of endogenous NEDD4L enhanced xCT activity in ESCC cells. NEDD4L inactivation desensitized ESCC cells to ferroptosis through upregulating xCT function and loss of NEDD4L stimulated tumor growth in xenograft mouse models. In addition, we uncovered that NEDD4L directly bonded to the ∆CT domain of xCT through its WW and HECT domain. Of note, we further elucidated that NEDD4L served as a bridge to transfer the attached ubiquitin molecules to xCT and enhancing the degradation of xCT through K48-linked polyubiquitination, leading to the promotion of ferroptosis and ultimately suppressing ESCC cell growth. Collectively, we identify an E3 ligase NEDD4L is crucial in governing the stability of xCT and mediating ferroptosis in ESCC.

## Results

### NEDD4L expression is low and serves as a good prognosis marker in ESCC

**Fig. 1:**
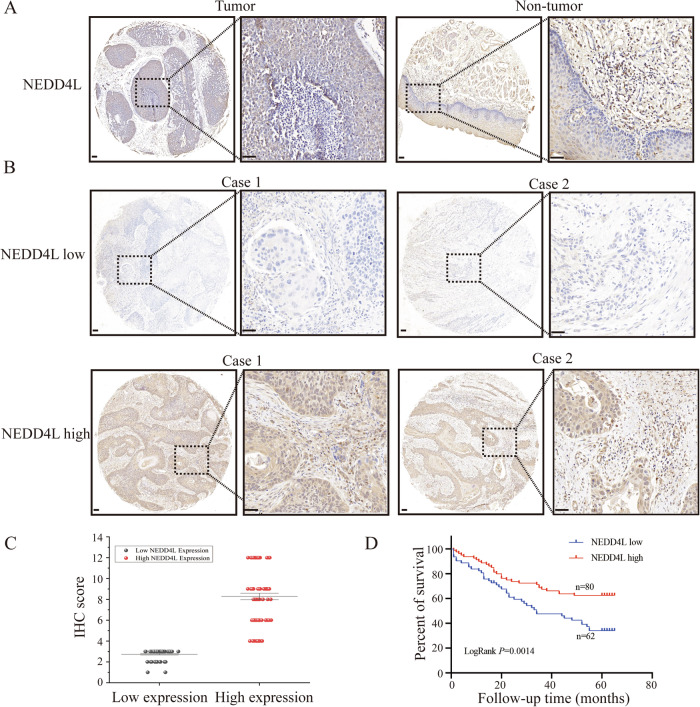
NEDD4L expression is low and serves as a good prognosis marker in ESCC. **A** NEDD4L protein expression was decreased in ESCC tissues (*n* = 25) compared with normal tissue (*n* = 25) as analyzed by IHC. Scale bars, 100 μm. **B** Representative images of NEDD4L low expression (*n* = 62) and high expression (*n* = 80) analyzed by IHC. Scale bars, 100 μm. **C** NEDD4L expression score in 142 cases of ESCC (*n* = 142). **D** Overall survival analysis revealed that higher expression of NEDD4L was related with poorer overall survival of ESCC patients (*n* = 142). *p* <0.01, log-rank test.

The analysis results from TCGA database (https://tcga-data.nci.nih.gov/tcga/) implied that NEDD4L mRNA in esophageal cancer tissue is reduced compared with normal esophageal tissue (Fig. S2A). However, the data obtained from GEPIA database (http://gepia.cancer-pku.cn/) showed an increase of NEDD4L expression in esophageal cancer (Fig. S2B). To verify these findings at the protein level, we first examined the NEDD4L expression in ESCC clinical samples through IHC. In comparison to normal esophageal tissue, NEDD4L expression was significantly reduced in ESCC (Fig. 1A). We further performed IHC scoring on 142 cases of ESCC (Fig. 1B, C), and uncovered that NEDD4L expression negatively connected with tumor invasion (*p* < 0.05), lymph node metastasis (*p* < 0.05) and distant metastasis (*p* = 0.001) (Table 1). In addition, further analysis confirmed that low expression of NEDD4L (*p* < 0.05), invasion (*p* < 0.05) and lymph node metastasis (*p* < 0.05) are risk factors for poor prognosis (Table 2). Remarkably, a lower NEDD4L protein level strongly predicted poorer overall survival of ESCC patients (*p* < 0.01) (Fig. 1D).

**Table 1:** Clinicopathological correlation of NEDD4L expression in ESCC.

	Cases	NEDD4L expression		χ^2^	*P* value
		Low	High		
Age					
≤60	56	27	29	0.779	0.377
＞60	86	35	51		
Gender					
Male	90	39	51	0.011	0.917
Female	52	23	29		
Differentiation					
G1	69	27	42	1.133	0.568
G2	58	28	30		
G3	15	7	8		
Tumor invasion					
T1	28	9	19	14.899	**0.002**
T2	59	18	40		
T3	50	29	20		
T4	7	6	1		
Lymph node metastasis					
N0	101	38	63	5.185	**0.023**
N1	41	24	17		
Distant metastasis					
M0	126	49	77	10.357	**0.001**
M1	16	13	3		

Bold values identify statistical significance (*P* < 0.05).

**Table 2:** Cox proportional hazard regression analyses for overall survival.

	Univariable analysis		Multivariable analysis	
Clinical and molecular characteristics	HR(95%CI)	*P* value	HR(95%CI)	*P* value
Age	1.238 (0.771–1.988)	0.377		
Gender	1.515 (0.914–2.511)	0.107		
Differentiation	1.017 (0.637–1.626)	0.943		
Tumor invasion	0.421 (0.262–0.675)	**<0.001**	0.522 (0.310–0.878)	**0.014**
Lymph node metastasis	0.448 (0.277–0.726)	**0.001**	0.561 (0.325–0.968)	**0.038**
Distant metastasis	0.247 (0.136–0.449)	**<0.001**	0.552 (0.270–1.128)	0.103
NEDD4L expression	2.123 (1.320–3.413)	**0.002**	1.708 (1.003–2.908)	**0.049**

Bold values identify statistical significance (*P* < 0.05).

### NEDD4L inhibits ESCC progression in vivo and in vitro

**Fig. 2:**
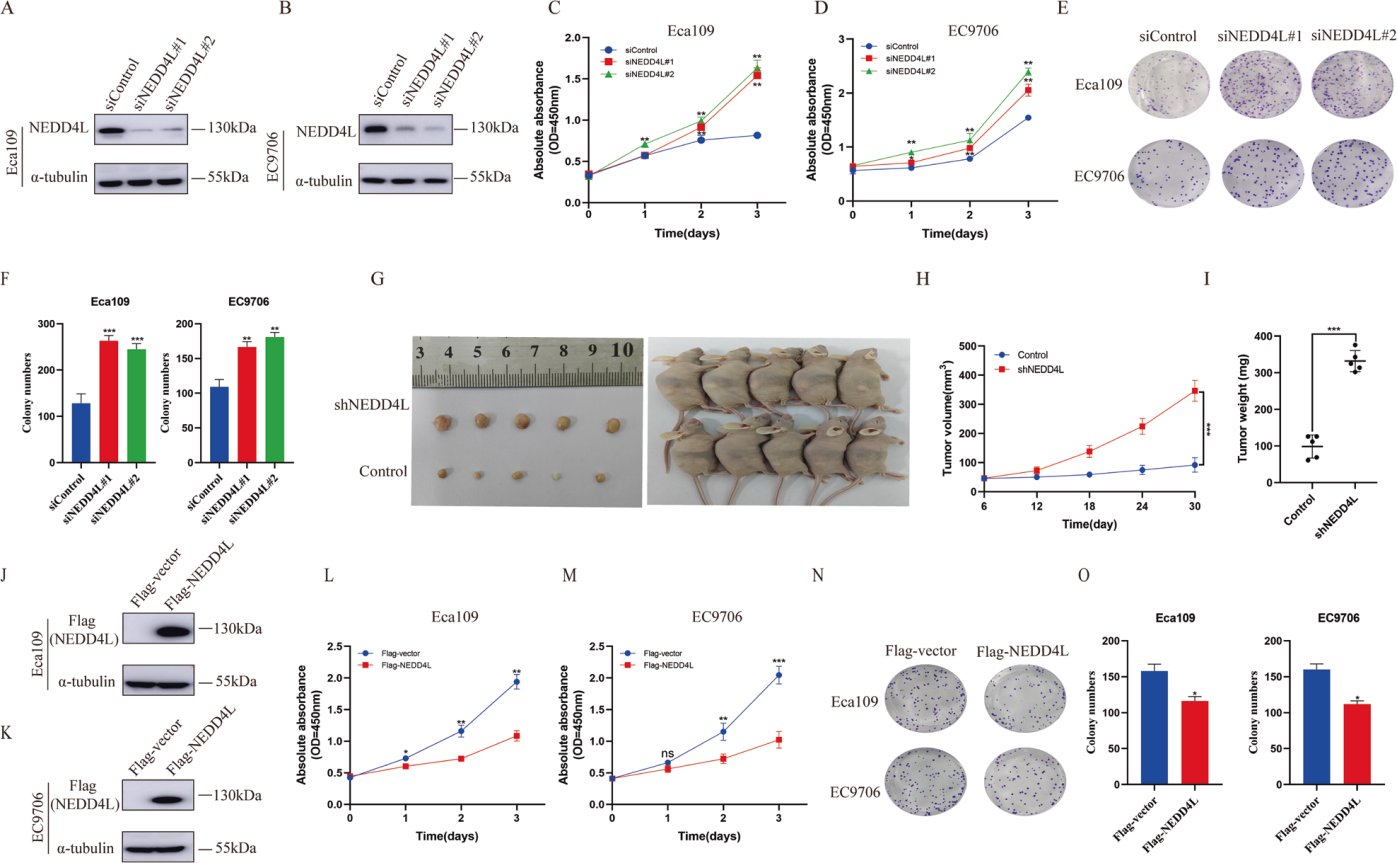
NEDD4L inhibits ESCC progression in vivo and in vitro. **A, B** NEDD4L silencing efficiency in ESCC cell lines. Eca109 and EC9706 cells were transfected with NEDD4L siRNAs. The silencing efficiency was measured via western blotting. **C, D** Silencing NEDD4L promoted the proliferation of ESCC cells. Eca109 and EC9706 were transfected with siControl or siNEDD4L. There were two different siRNAs be used. After 24 hours, the CCK8 was used to determine the cellar metabolic activity at indicated time points after infection (*n* = 3 per group). **E, F** Silencing NEDD4L increased the clone numbers of ESCC cells. Eca109 and EC9706 cells were transfected with indicated 50 nM siNEDD4L or siControl. Quantification of clone formation was shown at the indicated time points (n = 3 per group). **G**–**I** NEDD4L depletion promoted tumor growth of EC9706 cells in xenograft model. Female nude mice bearing EC9706 tumors were treated daily with control (*n* = 5) or shNEDD4L (*n* = 5) at the indicated concentrations. The growth of xenografts was monitored over 4 weeks. **J, K** NEDD4L overexpression efficiency in ESCC cell lines. Eca109 and EC9706 cells were transfected with NEDD4L plasmids. The overexpression efficiency was measured via western blotting. **L, M** Overexpression NEDD4L inhibited the proliferation of ESCC cells. Eca109 and EC9706 were transfected with Flag-NEDD4L or Flag-vector. CCK8 solution was added to determine the cellar metabolic activity at indicated time points (*n* = 3 per group). **N, O** Overexpression NEDD4L decreased the clone numbers of ESCC cells. Eca109 and EC9706 cells transfected with indicated 5 μg Flag-vector or Flag-NEDD4L plasmids. Quantification of clone formation was shown at the indicated time points (*n* = 3 per group). The data in **I** is presented as the mean ± SEMs. Statistical analysis was performed using Student’s *t*-test. *p < 0.05; **p < 0.01; ***p < 0.001. The other data are presented as the mean ± SD. Statistical significance was determined by one-way ANOVA. *p < 0.05; **p < 0.01; ***p < 0.001; ns, not significant.

To investigate the impact of NEDD4L on ESCC phenotypes, we silenced NEDD4L by two independent siRNAs in ESCC cells (Fig. 2A, B). Then, cell invasion and migration ability were determined by trans well assay and wound healing assay, respectively. The trans well assay indicated that silencing NEDD4L increased EC9706 cells invasion capacity (Fig. S3A, B). Wound healing assay revealed that NEDD4L depletion cells showed increased wound closure speed compared to control group (Fig. S3C, D). In addition, the CCK8 assay results showed that silencing NEDD4L significantly promoted the cell growth both in Eca109 and EC9706 (Fig. 2C, D), consistent with the results of clone formation assay (Fig. 2E, F). Subsequently, we established a stable NEDD4L silencing model in EC9706 cell line and evaluated the function of NEDD4L in vivo by xenograft mouse model. The results indicated that silencing NEDD4L increased tumor growth speed compared to the control group in vivo (Figs. S3E, and 2G-I).

Moreover, we overexpressed NEDD4L in ESCC cells to measure the influence of overexpressing NEDD4L on the invasion, migration and proliferation abilities of Eca109 and EC9706 cells (Fig. 2J, K). As shown in Fig. S3F–I, overexpression NEDD4L suppressed the invasion capacity and wound healing speed in EC9706 cells (Fig. S3F–I). Besides, CCK8 assay and clone formation assay confirmed that overexpression of NEDD4L attenuated cell proliferation and colony formation ability, respectively (Fig. 2L–O).

### NEDD4L promotes ferroptosis in ESCC

**Fig. 3:**
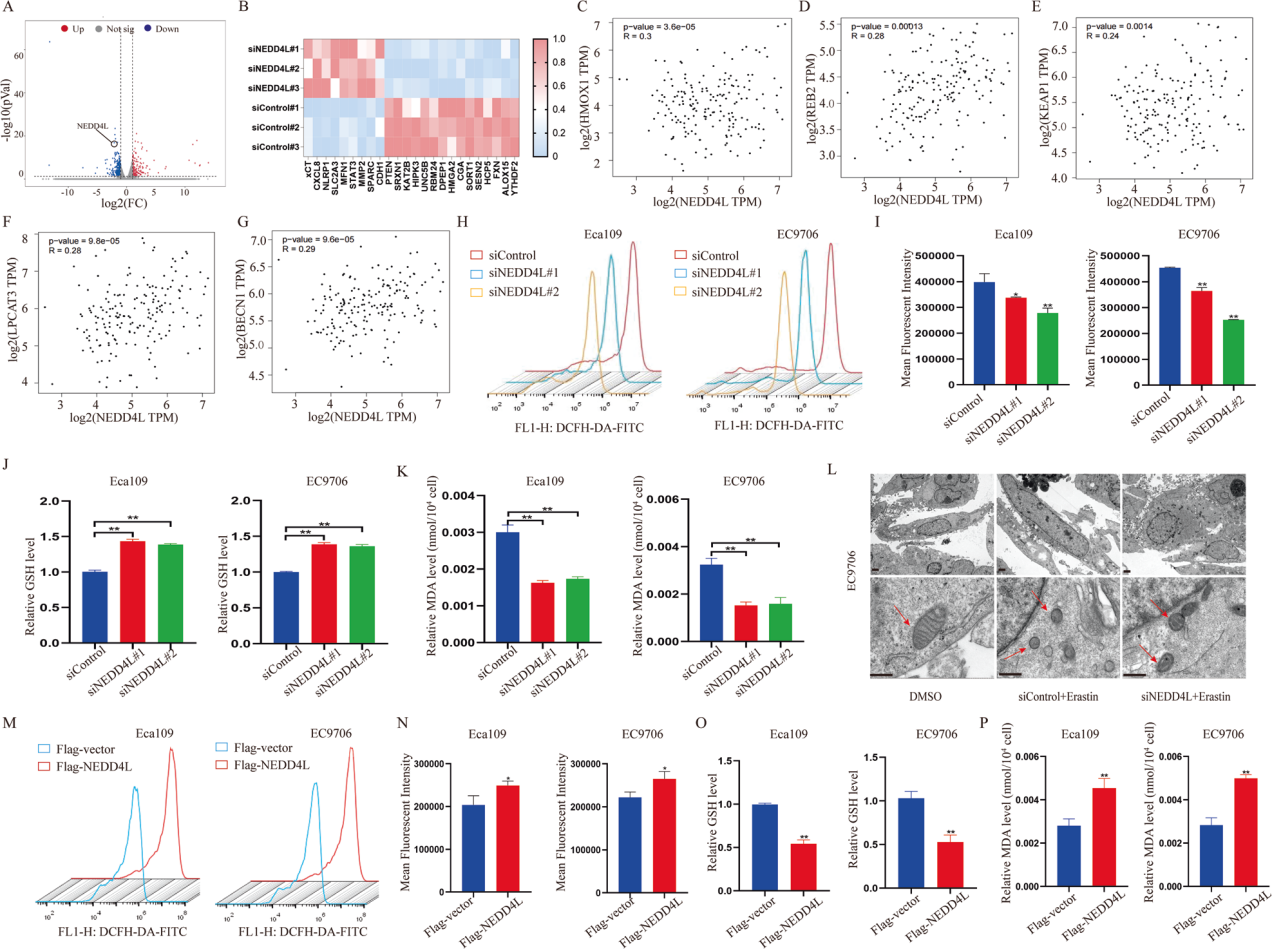
NEDD4L promotes ferroptosis in ESCC cells. **A** Volcano plot of significantly up and down-regulated genes after NEDD4L depletion. **B** Heat map of mRNA changes in siControl (*n* = 3) and siNEDD4L (*n* = 3) of EC9706 by bulk RNA-seq. **C**–**G** Publicly available data showed that NEDD4L expression is positively correlated with that of the ferroptosis related genes HMOX1, IREB2, KEAP1, LPCAT3 and BECN1 in esophageal cancer (http://gepia.cancer-pku.cn/). **H, I** ROS level in Eca109 and EC9706 cells transfected with siControl/siNEDD4L for 48 h were detected. *n* = 3 per group. **J** Silencing NEDD4L promoted GSH level in ESCC cells. Eca109 and EC9706 cells were transfected with siControl/siNEDD4L for 48 h. GSH level was determined at 412 nm. *n* = 3 per group. **K** Silencing NEDD4L reduced MDA content in ESCC cells. Eca109 and EC9706 cells were transfected with siControl/siNEDD4L 48 h. MDA content was measured at 532 nm and 600 nm. *n* = 3 per group. **L** Representative cell and mitochondrial ultrastructural images of EC9706 cells transfected with siContrlo/siNEDD4L under the treatment of erastin for 24 h. Scale bar = 5 μm. **M, N** Overexpressing NEDD4L promoted ROS level in ESCC cells. Eca109 and EC9706 cells were transfected with Flag-vector or Flag-NEDD4L plasmids for 48 h. *n* = 3 per group. **O** Overexpressing NEDD4L decreased GSH level in ESCC cells. Eca109 and EC9706 cells transfected with Flag-vector or Flag-NEDD4L plasmids for 48 h. *n* = 3 per group. **P** Overexpressing NEDD4L increased MDA content in ESCC cells. Eca109 and EC9706 cells transfected with Flag-vector or Flag-NEDD4L plasmids for 48 h. *n* = 3 per group. The data are presented as the mean ± SD. Statistical significance in **I–K** were determined by one-way ANOVA. *p < 0.05; **p < 0.01; ***p < 0.001. Statistical significance in **N–P** Statistical analysis were performed using Student’s *t*-test. *p < 0.05; **p < 0.01; ***p < 0.001.

Ferroptosis has been suggested to act as a tumor-suppressive mechanism in ESCC [20, 21]. To explore the special role of NEDD4L in ferroptosis, we performed RNA seq analysis on both siControl and siNEDD4L cells. As shown in Fig. 3A, 207 genes were up-regulated, and 397 genes were down-regulated in the absence of NEDD4L (Fig. 3A). Notably, the heatmap results revealed that multiple genes associated with ferroptosis were activated by silencing NEDD4L, especially xCT, arachidonic acid 15-lipoxygenase-1 (ALOX15), sestrin2 (SESN2) and phosphatase and tensin homolog (PTEN) (Fig. 3B). In addition, by analyzing public databases, it was found that NEDD4L expression level was positively related to a series of ferroptosis target genes, including HMOX1 [22], IREB2 [23], KEAP1 [24], LPCAT3 [25] and BECN1 [26] (Fig. 3C–G). These results suggested a potential link between NEDD4L and ferroptosis.

Therefore, we further explored the characteristic phenotypes of ferroptosis in the absence of NEDD4L. Given that the excess ROS is the core features of ferroptosis, we first detected ROS levels after silencing NEDD4L. The ROS assay revealed that depletion of NEDD4L inhibited the accumulation of ROS both in Eca109 and EC9706 cells (Fig. 3H, I). Besides, GSH and MDA content are essential indexes of ferroptosis. Interestingly, we also observed that the GSH level was increased, while the MDA level was decreased in NEDD4L-silenced ESCC cells by using microplate reader (Fig. 3J, K). Afterwards, we introduced a ferroptosis inducer, erastin to investigate the effect of NEDD4L deletion on ferroptosis. Flow cytometry results indicated that silencing NEDD4L decreased erastin-induced cell death in Eca109 and EC9706 cells (Fig. S4A–D). Interestingly, using transmission electron microscopy, we observed that cells treated with erastin displayed mitochondrial shrinkage, characterized by disappearing mitochondrial ridge and increasing membrane density. However, this effect could be partially attenuated by NEDD4L depletion (Fig. 3L). In addition, a decrease in red fluorescence and a concurrent increase in green fluorescence in JC-1 dye-stained cells are indicative of a reduction in MMP. Thus, we measured the changes of MMP by using optical microscopy after transfecting with siNEDD4L for 48 hours. As shown in Fig. S4E, F, the green fluorescence was decreased in the group of siNEDD4L, suggesting an increase in the MMP of ESCC cells after silencing NEDD4L under the treatment of erastin (Fig. S4E, F).

Correspondingly, key indicators of ferroptosis include ROS level, GSH level and MDA content after overexpression of NEDD4L were assessed. As expected, overexpression NEDD4L could promote ROS level and MDA content, but restrain the GSH level both in Eca109 and EC9706 cells (Fig. 3M–P). Moreover, we detected the expression of NEDD4L in multiple ESCC cell lines (Fig. S4G) and overexpressed NEDD4L in the KYSE30 cells with the lowest expression to verify the effect of NEDD4L on ferroptosis. The results showed that overexpression of NEDD4L significantly improved ROS and MDA level, while inhibited GSH level in KYSE30 cells (Fig. S4H–J).

### NEDD4L expression reversely relates with xCT level in ESCC

**Fig. 4:**
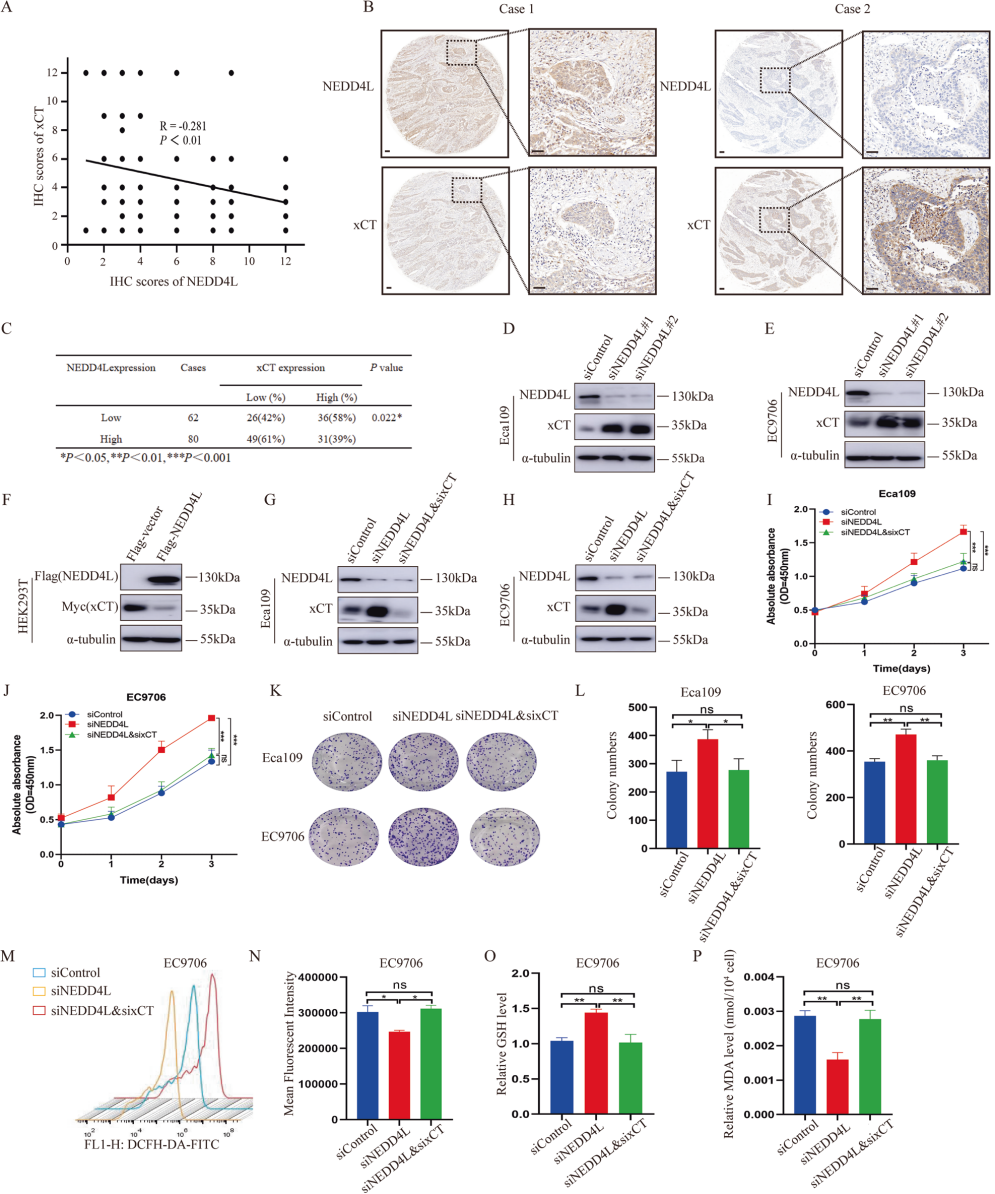
NEDD4L expression reversely relates with xCT level in ESCC. **A**–**C** Low NEDD4L expression was correlated with an increase level of xCT in ESCC samples (*n* = 142). **D, E** Silencing NEDD4L increased the xCT protein level in Eca109 and EC9706 cells. **F** NEDD4L overexpression reduced xCT protein level in HEK293T cells. **G, H** Silencing of xCT reduced NEDD4L depletion resulting in an increase in xCT protein levels. **I, J** Silencing xCT rescued the proliferation ability of cells with the NEDD4L depletion. *n* = 3 per group. **K, L** Silencing xCT rescued clone numbers with the NEDD4L depletion. *n* = 3 per group. **M, N** Silencing xCT rescued ROS level with the NEDD4L depletion. *n* = 3 per group. **O** Silencing xCT rescued GSH level with the NEDD4L depletion. *n* = 3 per group. **P** Silencing xCT rescued MDA content with the NEDD4L depletion. *n* = 3 per group. The data are presented as the mean ± SD. Statistical significance was determined by one-way ANOVA. *p < 0.05; **p < 0.01; ***p < 0.001; ns, not significant.

Considering NEDD4L is the E3 ubiquitin ligase which has the highest confidence connect with xCT (Fig. S1 and Table S2) and xCT expression is increased in ESCC samples when compared to normal tissue (Fig. S5A) (UALCAN database: https://ualcan.path.uab.edu/index. html). Then, we performed xCT expression scoring on 142 cases of ESCC and investigated the relationship between NEDD4L and xCT expression in clinical samples (Figs. 4A and S5B–D). Interestingly, the findings indicated a negative correlation between the expression level of NEDD4L and xCT (*p* < 0.05) (Fig. 4B, C). Subsequently, we clarified the relationship between NEDD4L and xCT at protein level. As shown in Fig. 4D, E, silencing NEDD4L could promote xCT protein level in both Eca109 and EC9706 cells (Fig. 4D, E). Correspondingly, NEDD4L overexpression inhibited xCT protein level in HEK293T cells (Fig. 4F).

Additionally, we constructed xCT siRNA for serval rescue assays (Fig. S5E, F). The results showed that silencing NEDD4L in Eca109 and EC9706 cells could promote xCT protein expression, cell proliferation and clone number, whereas further knockdown of xCT could partially rescue these effects (Fig. 4G–L). Notably, silencing xCT reversed the reduction of ROS and MDA levels, as well as the increase of GSH level caused by NEDD4L knock down both in Eca109 and EC9706 cells. These findings suggested that the regulation of ferroptosis by NEDD4L in ESCC may be achieved through targeting xCT (Fig. 4M–P).

### NEDD4L interacts with ∆CT domain of xCT through its WW and HECT domain

**Fig. 5:**
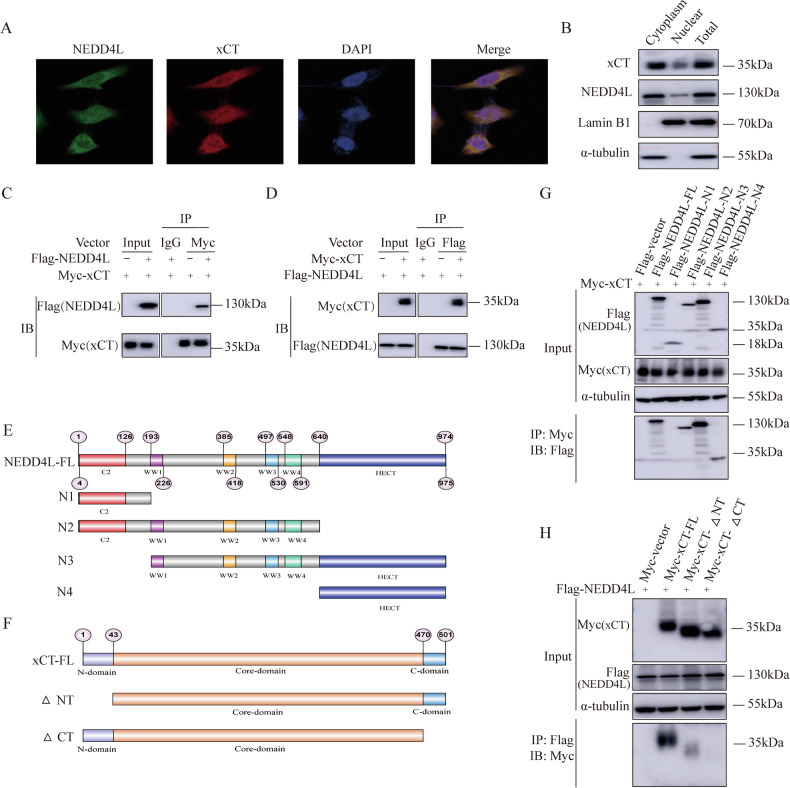
NEDD4L associates with ∆CT domain of xCT through its WW and HECT domain. **A** Intracellular localization of NEDD4L and xCT analyzed by IF assay. EC9706 cells were cultured in normal medium before fixation. Intracellular localization of xCT (red) and NEDD4L (green) were shown. Nuclei (blue) were stained with 4’,6-diamidino-2-phenylindole (DAPI). **B** Cytoplasm and nuclear were separated by kit. NEDD4L is mainly localized in the cytoplasm. α-tubulin and lamin B1 were used for cytoplasm and nuclear control. NEDD4L interacted with xCT in cytoplasm. **C, D** Co-IP assay revealed that NEDD4L connected with xCT in EC9706 cells. EC9706 cells were transfected with indicated Flag-NEDD4L and Myc-xCT plasmids, followed by Co-IP and western blotting detection. **E, F** NEDD4L and xCT domain structure and deletion mutants used for Co-IP assays. **G** NEDD4L bound to xCT at its WW and HECT domain. HEK293T cells were transfected with the indicated NEDD4L and xCT constructs, followed by Co-IP and western blotting assays. The WW and HECT domain of NEDD4L interacted with xCT. **H** xCT bonded to NEDD4L at its ∆CT domain. HEK293T cells were transfected with the indicated xCT and NEDD4L constructs, followed by Co-IP and western blotting detection.

In order to investigate the specific mechanism between NEDD4L and xCT in ESCC, we explored the localization of NEDD4L and xCT in ESCC cells. Immunofluorescence analysis results verified that NEDD4L interacted with xCT in cytoplasm in EC9706 cells (Fig. 5A). Similar results were confirmed by nucleocytoplasmic separation assay (Fig. 5B). Subsequently, immuno-precipitation (IP) analysis found that NEDD4L connected with xCT in EC9706 cells (Fig. 5C, D). To determine the specific domains for the interaction between NEDD4L and xCT, the corresponding deletion constructs were constructed and the associated domains was further clarified (Fig. 5E, F). The results confirmed that WW and HECT domain of NEDD4L was necessary domains for the interaction with xCT, and ∆CT domain was required for xCT to connect with NEDD4L (Fig. 5G, H).

### NEDD4L modulates xCT stability in ubiquitination dependent manner

**Fig. 6:**
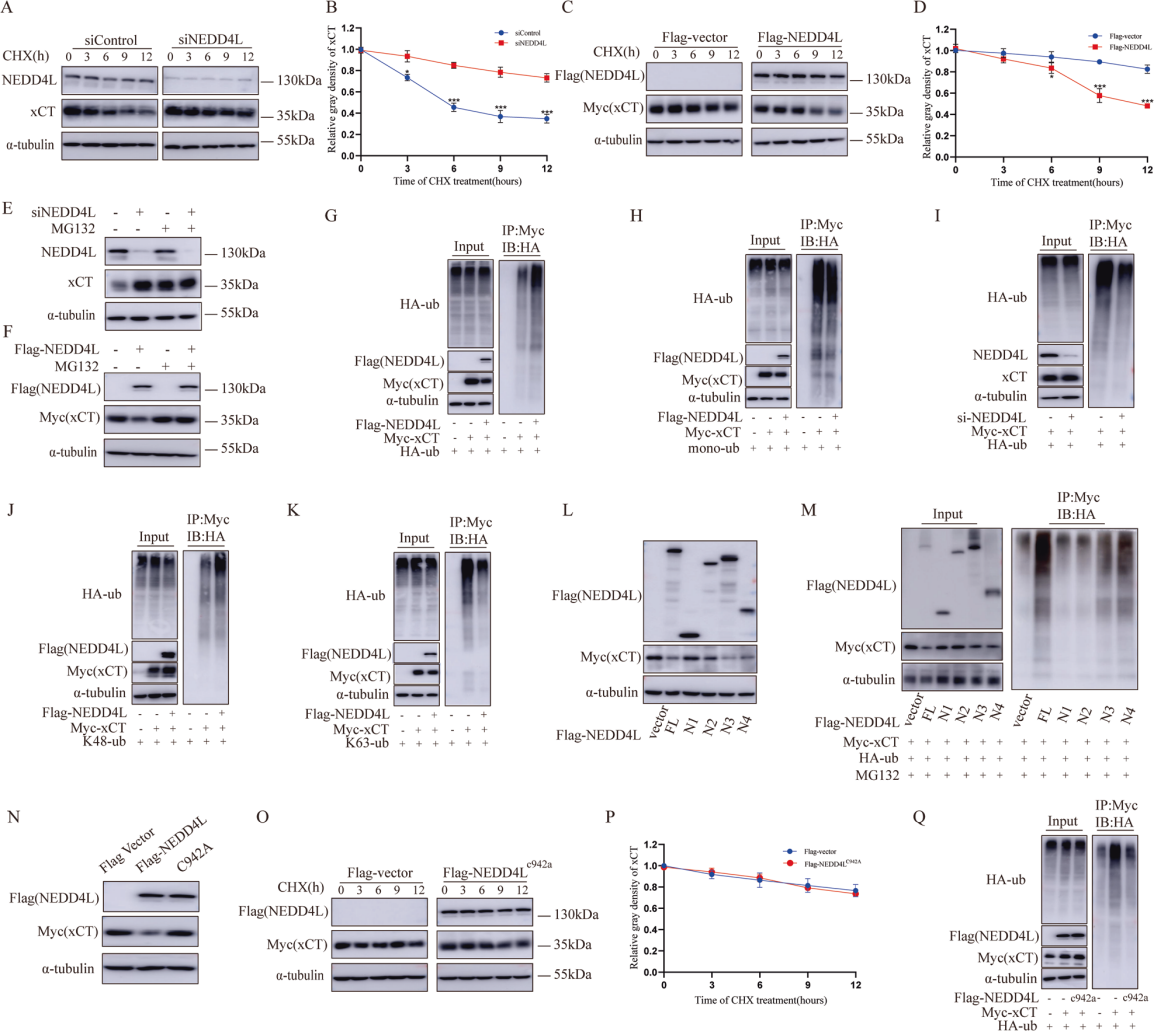
NEDD4L modulates xCT stability in ubiquitination dependent manner. Silencing NEDD4L decreased xCT half-life in EC9706 cells (**A, B**) and NEDD4L overexpression prolonged xCT half-life in HEK293T cells (**C, D**). The cells were treated with 100 μmol/L CHX for indicated time periods before being collected for western blotting assay. *n* = 3 per group. **E, F** NEDD4L upregulated xCT through proteosome. NEDD4L overexpression could inhibit xCT protein level and NEDD4L depletion could promote xCT protein level, which effect could be diminished by MG132. **G** Ubiquitin-based IP assays showed that overexpression NEDD4L promoted xCT overall poly-ubiquitination in HEK293T cells. **H** Ubiquitin-based IP assays showed that NEDD4L failed to facilitate xCT mono-ubiquitinaiton in HEK293T cells. **I** Silencing NEDD4L inhibited xCT overall poly-ubiquitination in EC9706 cells. **J** NEDD4L promoted xCT K48-linked ubiquitinaiton in HEK293T cells. **K** Ubiquitin-based IP assays showed that NEDD4L inhibited xCT K63-linked ubiquitinaiton in HEK293T cells. **L** The HECT domain of NEDD4L was necessary for NEDD4L to xCT protein suppression. **M** The HECT domain of NEDD4L was necessary for NEDD4L to regulate ubiquitination of xCT. **N** Mutations in NEDD4L that disrupted its ubiquitination activity compromised NEDD4L’s capacity to degrade the xCT protein. **O, P** HEK293T cells were transfected with Flag-tag or Flag NEDD4L^c942a^ and Myc-xCT plasmids. The cells were treated with 100 μmol/L CHX for indicated time periods before being collected for western blotting assays. *n* = 3 per group. **Q** The effects of expression of Flag NEDD4L and its mutants on ubiquitination of Myc- xCT in HEK293T cells detected by Ubiquitin-based IP assays. The data are presented as the mean ± SD. Statistical significance was determined by two-way ANOVA. *p < 0.05; **p < 0.01; ***p < 0.001.

Given the role of NEDD4L E3 ubiquitin ligase, we initially examined the effect of silencing NEDD4L on xCT stability by cycloheximide (CHX). Indeed, silencing of NEDD4L extended the half-life of xCT, while overexpression of NEDD4L has the opposite effect (Fig. 6A–D). Moreover, the inhibition effect of xCT protein by NEDD4L overexpression and the promotion effect of xCT protein by NEDD4L depletion can be attenuated by MG132 (proteasome inhibitor), suggesting that the regulation between NEDD4L and xCT was proteasome dependent (Fig. 6E, F). Therefore, the impact of silencing NEDD4L on xCT ubiquitination was further detected. The results indicated that NEDD4L significantly enhanced the poly-ubiquitination rather than mono-ubiquitination level of xCT (Fig. 6G, H). Next, we explored the influence of silencing NEDD4L on the overall ubiquitination level of xCT and the results suggested that NEDD4L depletion significantly decreased endogenous xCT ubiquitination in EC9706 cells (Fig. 6I). Ubiquitin contains seven lysine residues (K6, K11, K27, K29, K33, K48, and K63) [27, 28]. The specific lysine residues within the target protein can determine the type and function of the ubiquitin modification. For example, ubiquitin chains linked through K48 typically target proteins for degradation by the proteasome, while chains linked through lysine K63 are often involved in signaling processes, such as DNA repair, endocytosis, and immune response [28, 29]. Our results proved that NEDD4L depletion significantly increased K48-linked ubiquitination of xCT, while decreased K63-linked ubiquitination of xCT in HEK293T cells (Fig. 6J, K). We further verified the functional domain of NEDD4L in inducing xCT poly-ubiquitination. Figure 6L, M showed that the HECT domain of NEDD4L is required for xCT protein degradation and poly-ubiquitination (Fig. 6L, M).

Subsequently, we mutated the C942A site of NEDD4L to eliminate the E3 ligase activity of NEDD4L [30, 31]. The results implied that the NEDD4L^C942A^ mutant, which lacks catalytic activity, was unable to affect xCT expression or half-life (Fig. 6N, P). Besides, ubiquitin-based IP assays showed that NEDD4L^C942A^ mutant could not stimulate ubiquitination of xCT in HEK293T cells (Fig. 6Q). These findings indicated that the impact of NEDD4L on facilitating xCT ubiquitination depends on its E3 ligase activity.

## Discussion

**Fig. 7:**
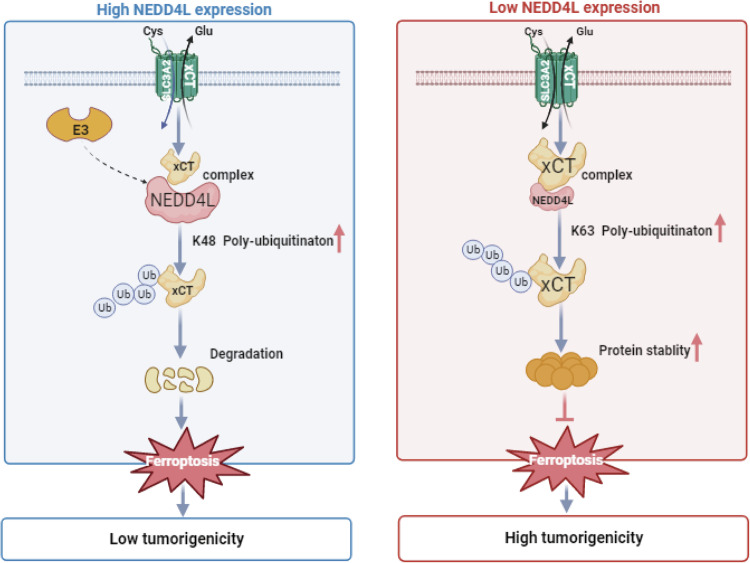
NEDD4L interacts with xCT, promotes xCT K48-linked ubiquitination and degradation, which activates the ferroptosis and inhibits ESCC cancer progression.

Ferroptosis plays a crucial role in drug resistance, cancer progression and immunity through multiple PTMs, including ubiquitination, methylation, acetylation and phosphorylation [8, 32]. Ubiquitination, which is closely related to protein stability and degradation, has been a topic of great interest in the field of cancer research among various PTMs. In the present study, we have demonstrated E3 ubiquitin ligase NEDD4L as a bona fide regulator of xCT in ESCC. NEDD4L is low expressed, while xCT is overexpressed in human ESCC samples. Besides, we revealed a novel mechanism by which NEDD4L regulates ESCC cell growth via targeting xCT. Notably, we identified that NEDD4L promotes ferroptosis by catalyzing proteasome dependent degradation and K48-linked poly-ubiquitination of xCT in ESCC (Fig. 7). In this regard, we speculate that the enhancement of NEDD4L, which leads to the suppression of xCT and activation of ferroptosis pathway may be a potential therapeutic target for ESCC patients.

In contrast to prior research perspectives that NEDD4L acts as a tumor suppressor, recent studies have implicated it also acts as an oncogene in certain contexts [33, 34]. For example, it is reported that the expression of NEDD4L was elevated in both cutaneous melanoma and lymph node metastatic melanoma compared to normal tissue. High level of NEDD4L may contribute to the promotion of melanoma cell growth in vivo [34]. Here, the results from our clinical data showed that NEDD4L expression is low and associated with tumor invasion, lymph node metastasis and distant metastasis. Moreover, we uncovered that NEDD4L depletion inhibited ferroptosis and promoted cell proliferation, which subsequently promoted cancer progression in vivo and in vitro. Consistent with our research findings, previous evidence showed that NEDD4L inhibited cell viability, cell cycle progression in ESCC [35]. Nevertheless, the dual role of NEDD4L in human cancer needs to be further clarified. Additionally, a group of E3 ubiquitin ligases, including HECTD3 [36], MIB1 [37] and FBW7 [38] have been reported to inhibit tumor growth by targeting different ferroptosis related genes. It is necessary to explore whether NEDD4L could suppress tumor progression via targeting other ferroptosis core genes, such as GPX4, ASCL4 and FTH1. Simultaneously, in light of NEDD4L could reduce cisplatin resistance and induce apoptosis in bladder cancer [39], exploring the role of NEDD4L in the drug resistance through ferroptosis will open novel avenue for human cancer treatment strategies. On the other hand, there is still no evidence showing that NEDD4L directly decreases drug resistance. Further study is essential and worthwhile to clarify whether NEDD4L can be applied in clinical trials for cancer therapy.

In addition, our study proved that silencing NEDD4L prevented the accumulation of ROS. However, ROS could be produced by mitochondria with the help of electrons and molecular oxygen, leading to an amplification of cellular oxidative stress reactions, along with the consequent harm to lipids, proteins and DNA [40, 41]. Consequently, an excess of ROS can also impact mitochondrial function. Previous study found that leucine-rich repeat kinase 2 (LRRK2) hindered ferroptosis induced by erastin, diminished ROS levels, and promoted the integrity of mitochondria [42]. Our results further elucidated that silencing NEDD4L lead to an increase MMP under the treatment of erastin. In addition, mitochondria shrink, reduced ridges and increased membrane density caused by erastin treatment could be partly reversed by NEDD4L depletion. Besides, our study also confirmed that in NEDD4L-silenced cells, GSH content was promoted, while MDA level was decreased. Based on these results, we suspected that ferroptosis is strongly linked with NEDD4L. It is worth noting that our heat map data demonstrated that the absence of NEDD4L significantly upregulated a cluster of genes associated with ferroptosis, such as ALOX15, SESN2, and PTEN. This finding indicated a potential regulatory role of NEDD4L in modulating the expression of these genes, which are known to play essential roles in regulating cellular sensitivity to ferroptosis [43–45]. Thus, it will be valuable for future research to identify which transcription factors influence this transcriptional process and how NEDD4L regulates ferroptosis-related genes transcription through modulating upstream or downstream factors. Furthermore, we also observed a positive correlation between the expression of NEDD4L and several ferroptosis-regulated target genes (HMOX1, IREB2, KEAP1, LPCAT3, and BECN1) through analysis of public databases. This correlation strengthened the hypothesis that NEDD4L may intricately regulate the ferroptotic process by influencing the expression of these genes, potentially serving as a key modulator or facilitator of ferroptosis in cellular contexts. Consequently, understanding the intricate mechanisms involving NEDD4L and its impact on ferroptosis, targeting NEDD4L or its downstream pathways associated with ferroptosis may hold promise for developing more effective treatment strategies for ESCC. Thus, further studies are warranted to elucidate the precise molecular mechanisms by which NEDD4L influences ferroptosis and its implications for cancer progression. Additionally, investigating potential therapeutic interventions that target NEDD4L or its downstream effectors could pave the way for novel treatments in cancer therapy, not limited to ESCC but potentially applicable to other cancers with dysregulated ferroptosis pathways. Meanwhile, due to the interaction between ferroptosis and other types of cell death, including autophagy and apoptosis [8, 46]. It is valuable to clarify whether there are other forms of cell death involved in the process of ESCC progression regulated by NEDD4L.

Increasing evidence suggested that xCT has the potential to serve as a biomarker in human cancers that play a crucial role in the development of tumors [47, 48]. For instance, previous studies have demonstrated that xCT can act as an oncogene in cancer cell growth by promoting cystine uptake and protecting cancer cells from oxidative stress and ferroptosis [49, 50]. However, the underlying regulatory and specific mechanism of xCT in human cancers remains largely unknown. In this study, we uncovered a novel regulatory pathway that modulated the sensitivity of tumor cells to ferroptosis by governing the protein stability of xCT. The expression of NEDD4L showed an inverse correlation with the level of xCT in ESCC samples. In addition, silencing xCT could rescue ROS, MDA reduction and GSH increase caused by NEDD4L deletion in ESCC cells. Mechanistically, NEDD4L interacted with ∆CT domain of xCT through its WW and HECT domain. Interestingly, inhibition of NEDD4L led to destabilization of xCT, subsequent diminished the susceptibility of ESCC cells to ferroptosis and promoted tumor growth. On the contrary, a recent study reported that homologous to the E6-associated protein carboxyl terminus domain containing 3 (HECTD3) promoted xCT degradation and stability via poly-ubiquitination manner, thereby triggering ferroptosis and suppressing tumor growth in colorectal cancer (CRC) [36]. Bearing this notion in mind, it would be interesting to determine why different E3 ubiquitin ligases have opposite effects via targeting xCT in human cancers. It would be also intriguing to screen many more E3 ubiquitin ligases targeting xCT for anticancer therapy. In addition, an earlier study implied that xCT is also required for the OTU domain-containing ubiquitin aldehyde-binding protein 1 mediated de ubiquitination, thereby inhibiting ferroptosis and promoting tumor growth in mice [51]. Therefore, it is worthwhile to further explore the relationship between de ubiquitination ligase and xCT.

Despite the prevailing view that xCT as an oncogene, Yan et al. have indicated that xCT has the potential to impede tumor metastasis under specific conditions. Although elevated xCT expression can enhance the growth of primary tumors, it was observed that metastasis was inhibited both in vivo and in vitro. This is probably due to the fact that cancer cells with high levels of xCT and undergo metastasis are especially vulnerable to oxidative stress [50]. In view of this, further investigation is urgently needed to clarify the role of high expression xCT in human cancers. Moreover, in pancreatic cancer, previous study confirmed that phosphorylation at S90/93/96 facilitates the binding of beclin 1 to xCT, resulting lipid peroxidation and ferroptosis, eventually extending the survival time of mice [26]. Additionally, it is reported that p53 exerted tumor suppressive effect by regulating xCT, and acetylation modification is indispensable in this process [52]. More importantly, the obesity-associated protein (FTO) can potentially suppress the progression of thyroid cancer by reducing the expression of xCT through N6 methyladenosine (m6A) modification [53]. Hence, the identification of other types of PTMs connection with xCT will be necessary for clarifying its roles in human cancers.

In summary, our study demonstrated that NEDD4L played an essential role in controlling the stability of xCT via ubiquitination in ESCC. NEDD4L was low expressed in human ESCC samples, silencing NEDD4L inhibited ferroptosis by stabilizing xCT, resulting in tumor growth promotion in xenograft mice models. Thus, as a novel modulator of xCT/ferroptosis signaling pathway, interfering with NEDD4L function or expression could be potential therapeutic strategies for ESCC.

## Materials and methods

### Clinical ESCC samples and immunohistochemistry (IHC) staining

142 cases of ESCC samples were obtained from the First Affiliated Hospital of Xinxiang Medical University and this study was approved by the Ethics Committee of the Xinxiang Medical University (XYLL-20220488). The pathological grade plus lymph node metastasis were checked by pathological specialists. The study participants did not receive preoperative treatment. All patients signed informed consent. Antibody against NEDD4L (ab46521, Abcam) and antibody against xCT (26864-1-AP, Proteintech) were used to IHC staining.

The IHC results were evaluated by a professional investigator blind to sample information. The staining intensity was evaluated on a scale from 0 to 3, where a score of 0 indicates absence of staining, and 3 indicates the most robust staining. Three randomly selected fields of each sample were observed under a 200X magnification to determine the percentage of cells displaying positive staining. The average percentage of positive staining was translated into score based on the following categories: 0 (0% to 25%), 1 (26% to 50%), 2 (51% to 75%), 3 (76% to 100%). The final IHC score was derived by adding the intensity score to the percentage score. For statistical analysis, ESCC cases were divided into NEDD4L or xCT high expression group (score ≥ 4) and low expression group (score < 4).

### Cell culture

EC9706, Eca109 and HEK293T cells were purchased form American Type Culture Collection (ATCC), KYSE30, KYSE140, KYSE410, KYSE450 were generously provided by Henan Key Laboratory of Tumor Molecular Therapy Medicine, China. Cells were maintained at 37 °C with 5% CO_2_. EC9706, Eca109, KYSE30, KYSE140, KYSE410 and KYSE450 cells are maintained with RPMI-1640 (SH30809.01, HyClone) supplemented with 2.05 mM L-glutamine and 10% fetal bovine serum (10270, Life Technologies), while HEK293T cells are incubated in DMEM medium (SH30022.01, HyClone) supplemented with 10% fetal bovine serum. Short Tandem Repeat (STR) was used for cell line authentication, and the results were consistent with STR data in ATCC and China Infrastructure of Cell Line Resources.

### Western blotting

The proteins were transferred to the PVDF membranes via electrophoresis. The antibodies used in this study are as follows: Anti-NEDD4L (4013S, CST, 1:1000); Anti-xCT (12691S, CST, 1:1000); Anti-Flag (AB0008, Abways, 1:1000); Anti-Myc (2278 s, CST, 1:1000); Anti-HA (3724S, CST, 1:1000); Anti-α-tubulin (66031-1-Ig, Proteintech, 1:1000). Following being washed 3 times with PBS, the membrane was incubated with second antibodies Goat Anti-Rabbit IgG (AB0101, Abways, 1:1000) or Goat Anti-Mouse IgG (AB0102, Abways, 1:1000). ECL kit (Millipore Corporation, Billerica) was employed to detect protein signals, while ECL system (Amersham Imager 600, GE) were used to capture fluorescent signals.

### Plasmids and siRNA

Flag NEDD4L^FL^ and NEDD4L^N1^, NEDD4L^N2^, NEDD4L^N3^, NEDD4L^N4^ mutants were constructed by cloning the cDNA of the full-length or truncated mutants into the CMV and EGFP sites of the pcDNA3.1-EF1a-mcs vector. Myc xCT, xCT^ΔCT^ and xCT^ΔNT^ were subcloned into pCDNA3.1-EGFP vector. The HA-Ub, HA-K48, and HA-K63 plasmids were donated by Henan Key Laboratory of Tumor Molecular Therapy Medicine. Small interfering RNAs were used to silencing NEDD4L and xCT. The NEDD4L and xCT siRNA sequences were shown in supplementary Table S1. Lipofectamine 2000 (1662298, Invitrogen) was used for plasmids transfection, while RNAiMAX (13778150, Invitrogen) was used for siRNA transfection.

### CCK8 assay

Cell Counting Kit-8 (CCK8) cell proliferation reagent (MC0301, Kermey) was used to measure cell proliferation. Eca109 and EC9706 cells were transfected with 50 nM siNEDD4L, sixCT for 48 h. Subsequently, the cell count was assessed, and a total of 5000 cells were distributed into 96-well plates. Then, 10 μL of CCK8 solution was added into cells and incubated for 2 hours in a 5% CO_2_ incubator at 37 °C. Absorbance at 450 nm (OD450) was measured at indicated time points.

### Clone formation assay

Eca109 and EC9706 cells were transfected with 50 nM NEDD4L or xCT siRNAs. The cells were trypsinized after 24 h of transfection, and subsequently a number of 500 cells per well was seeded in a 6-well plate. The culture medium was replaced every two days. Following two weeks of incubation, the colonies were gently washed three times with PBS and fixed with 4% paraformaldehyde for 10 minutes at room temperature. Finally, colonies were stained by crystal violet for 20 minutes.

### ROS assay

Highly Sensitive DCFH-DA-ROS assay kit (R252, Dojindo) was used to detect the ROS level according to the manufacturer’s instructions. Eca109, EC9706 and KYSE30 cells were transfected with 50 nM siNEDD4L/sixCT or 5 μg NEDD4L plasmid in 6-well plates. 48 h post-transfection, the culture medium was replaced with 2 mL working solution containing Highly Sensitive DCFH-DA Dye (1000-fold diluted) for 30 minutes at 37 °C incubator. Then the cells were collected and washed twice with HBSS. The changes in the levels of ROS were detected by flow cytometry (Beckman, America). The data was analyzed using Flowjo V10 software.

### Reduced glutathione (GSH) assay

GSH Detection Kit (BC1175, Solarbio) was used to measure the level of GSH. A number of 5 × 10^6^ Eca109, EC9706 or KYSE30 cells were transfected with 50 nM siNEDD4L/sixCT or 5 μg NEDD4L plasmid for 48 hours, followed by addition of 1 mL Reagent 1. After ultrasonic disruption of cells in an ice bath, cells were centrifuged at 12,000 g for 10 minutes, and the supernatant was collected. Then, a microplate reader was used to detect GSH level at 412 nm.

### Malondialdehyde (MDA) assay

The MDA level was assessed by MDA Content Detection Kit (BC0020, Solarbio) according to the manufacturer’s instructions. A number of 5 × 10^6^ Eca109, EC9706 or KYSE30 cells were transfected with 50 nM siNEDD4L/ sixCT or 5 μg NEDD4L plasmid for 48 hours. Subsequently, 1 mL extraction solution was added, followed by ultrasonic disruption of the cells. Cells were centrifuged at 8000 g for 10 minutes to collect the supernatant. The absorbance of the sample at 532 nm and 600 nm was measured. MDA content was calculated according to: MDA content (nmol/10^4^cell) = 32.258 × ΔA ÷ N × F

### Transmission electron microscopy (TEM)

EC9706 cells were transfected with 50 nM siNEDD4L or siControl for 24 hours, followed by treating with erastin (20 μM). After 24 h treatment, the cells were fixed by TEM fixative (G1102, Servicebio) for 30 minutes away from light at room temperature. Then, the cells were stored at 4 °C for transmission electron microscopy detection.

### RNA-sequencing (seq) and bioinformatics analysis

RNA-seq services were provided by LC Bio (Zhejiang, China). Briefly, total RNA was extracted from three sets of EC9706 cells with NEDD4L depletion (siNEDD4L) and three control (siControl) cells using the RNeasy kit from Qiagen. After that, the RNA samples underwent high-throughput RNA sequencing conducted by LC Bio (Zhejiang, China), followed by data analysis. Volcanic map analysis of differential gene expression was analyzed by LC Bio cloud platform (https://www.omicstudio.cn/login). Genes with fold change (FC) ≥ 2 or FC ≤ 1 and False Discovery Ratio adjusted *p* <0.05 were considered being differentially expressed. After that, differentially expressed genes associate with ferroptosis were normalized and heat map was drawn according to the gene expression.

### Immunofluorescence (IF) staining

A concentration of 4% paraformaldehyde was used to fix EC9706 cells for 15 minutes, followed by 0.2% Triton X-100 (T8787, Sigma) for 5 minutes of permeabilization, and then cells were blocked with 5% BSA for 1 h. Anti-NEDD4L mouse antibody (sc-514954, Santa Cruz) and anti-xCT rabbit antibody (26864-1-AP, Proteintech) were used overnight, followed by fluorescence conjugated secondary antibody (Invitrogen). Images were captured utilizing the Nikon A+ laser scanning confocal system while ensuring compliance with the Nyquist criterion. The obtained images were subsequently processed and compiled using Image J.

### Co-immunoprecipitation (Co-IP) assay

EC9706 and HEK293T cells were co-transfected with 5 μg Flag-NEDD4L plasmid (full length or domains) and 5 μg Myc-xCT plasmid. IgG (Santa Cruz) was used to pre-clear and then cell lysates were incubated together with Myc antibody (2278 s, CST), accompanied by rabbit IgG as the negative control. Flag antibody (AB0008, Abways) was adopted by western blotting for protein binding detection. 5 μg Myc-xCT plasmid (full-length or domains) was co-transfected with Flag-NEDD4L plasmid. IgG (Santa Cruz) was used to pre-clear and then cell lysates were incubated with Flag antibody (AB0008, Abways), accompanied by mouse IgG as the negative control. Western blotting together with Myc antibody (2278 s, CST) was used to detected bound proteins.

### Protein stability assays

HEK293T cells were transfected with 5 µg Flag-NEDD4L or Flag-vector plasmids, while EC9706 cells were transfected with 50 nM siNEDD4L or siControl in 24-well plates. Subsequently, a concentration of 100 µM CHX (C7698, Sigma) was added into HEK293T and EC9706 cells at indicated time points after 24 h and 36 h of transfection, respectively. Western blotting was used to visualize xCT protein degradation.

### Protein ubiquitination assays

Protein ubiquitination was detected as described in previous study [14]. Briefly, EC9706 and HEK293T cells were transfected with HA Ubi, K48 Ubi, and K63 Ubi plasmids, together with Myc-xCT and Flag-NEDD4L plasmids. 24 h post-transfection, cells were treated by MG132 (HY-13259, MCE) at a concentration of 10 µM for 12 h. Then, 20 µL of protein A (P2051, Beyotime) was added to pre-clear the total protein for 4 h, followed by the collection of supernatants. After that, Myc antibody were used for the immunoprecipitated, while western blotting together with HA antibody (3724S, CST) was used for the detection of the ubiquitination of xCT.

### Xenograft tumor model

EC9706 cells transfected with control or shNEDD4L lentiviral vectors were used to establish stable silencing cell line. Then, 10 female BALB/c nude mice (4-week-old) purchased from Beijing Vital River Laboratory Animal Technology were randomly and blindly divided into shControl and shNEDD4L (*n* = 5 per group). A number of 5 × 10^5^ cells together with Matrigel solution were injected into the right dorsal flank of the mice. Tumor size was measured every 6 days, and the progression of tumor formation was tracked in nude mice for a duration of 30 days. The experiments were conducted following protocols approved by the ethics committee at Xinxiang Medical University (XYLL-20220487).

### Statistical analysis

Data were presented as the mean ± SD. Differences between two independent groups were tested with student’s *t*-test. Variance is similar between comparison groups. Pearson χ^2^ test was used to determine the relationship between NEDD4L and clinicopathological characteristics. Survival hazard was assessed through the application of univariate and multivariate Cox proportional hazard regression models. Differences were statistically significant when *p* < 0.05 (*p < 0.05, **p < 0.01, ***p < 0.001).

## Supplementary information

Supplementary materials

## Data Availability

RNA-seq data have been deposited in the Gene Expression Omnibus under accession number GSE252141. All data in this study are available within the article and supplementary information or from the corresponding authors on reasonable request.
