# Supplementary material for: NEDD4L contributes to ferroptosis and cell growth inhibition in esophageal squamous cell carcinoma by facilitating xCT ubiquitination
Source: Cell Death Discov. 2024 Nov 18;10:473. doi: 10.1038/s41420-024-02243-5 (PMC11574128; doi:10.1038/s41420-024-02243-5)

# NEDD4L contributes to ferroptosis and cell growth inhibition in esophageal squamous cell carcinoma by facilitating xCT ubiquitination

Zhen Chen[1,2,3,4], Weilong Wang[1,2,3,4], Jinghan Hou[1,2,3,4], Can Gao[1,2,3,4], Meili Song[1,3,4], Zijun Zhao[1,3,4], Ruirui Guan[1,3,4], Jingsheng Chen[1,3,4], Huicheng Wu[1,3,4], Siti Razila Abdul Razak[2], Tao Han[1,4,5], Junbo Zhang[6], Lidong Wang[7], Nor Hazwani Ahmad[2*], Xiumin Li[1,3,4*]

## Supplementary results

## Supplementary Tables

**Table S1.** Small interfering RNA sequence for NEDD4L and xCT.

| | Sequence (5'-3') forward | Sequence (5'-3') reverse |
|---|---|---|
| siNEDD4L#1 | CCCUAUACAUUUAAGGACUdTdT | AGUCCUUAAAUGUAUAGGGdTdT |
| siNEDD4L#2 | GGAGAAUUAUGUCCGUGAAdTdT | UUCACGGACAUAAUUCUCCdTdT |
| sixCT | CCAGGUGGUUUAGAAUAAUTT | AUUAUUCUAAACCACCUGGTT |
| siControl | UUCUCCGAACGUGUCACGUTT | ACGUGACACGUUCGGAGAATT |

**Table S2.** E3 ligases interact with xCT

| E3 | E3GENE | SUB | PFAM | GO | NET | MOTIF | SCORE | Confidence interaction |
|---|---|---|---|---|---|---|---|---|
| Q96PU5 | NEDD4L | Q9UPY5 | 1.00 | 5.73 | 1.00 | 6.61 | 0.829 | High |
| Q86TM6 | SYVN1 | Q9UPY5 | 1.00 | 1.25 | 1.00 | 6.61 | 0.714 | Middle |
| Q86YT6 | MIB1 | Q9UPY5 | 1.00 | 2.33 | 1.00 | 2.80 | 0.693 | Middle |
| P46934 | NEDD4 | Q9UPY5 | 1.00 | 5.73 | 1.00 | 1.00 | 0.681 | Middle |
| P62879 | GNB2 | Q9UPY5 | 1.00 | 5.73 | 1.00 | 1.00 | 0.681 | Middle |
| P22681 | CBL | Q9UPY5 | 1.00 | 3.98 | 1.00 | 1.00 | 0.671 | Middle |
| Q9UKV5 | AMFR | Q9UPY5 | 1.00 | 1.51 | 1.00 | 2.80 | 0.652 | Low |
| P43034 | PAFAH1B1 | Q9UPY5 | 1.00 | 3.77 | 1.00 | 1.00 | 0.640 | Low |
| O14512 | SOCS7 | Q9UPY5 | 1.00 | 2.88 | 1.29 | 1.00 | 0.639 | Low |
| Q9ULV8 | CBLC | Q9UPY5 | 1.00 | 2.88 | 1.29 | 1.00 | 0.639 | Low |
| Q8TCQ1 | MARCH1 | Q9UPY5 | 1.00 | 1.25 | 1.00 | 2.80 | 0.633 | Low |
| Q8WY64 | MYLIP | Q9UPY5 | 1.00 | 1.25 | 1.00 | 2.80 | 0.633 | Low |
| Q5T0T0 | MARCH8 | Q9UPY5 | 1.00 | 1.25 | 1.00 | 2.80 | 0.633 | Low |
| P14373 | TRIM27 | Q9UPY5 | 1.00 | 1.51 | 1.00 | 2.12 | 0.624 | Low |
| Q99732 | LITAF | Q9UPY5 | 1.00 | 2.88 | 1.00 | 2.12 | 0.613 | Low |
| Q9P1Y6 | PHRF1 | Q9UPY5 | 1.00 | 2.88 | 1.00 | 2.12 | 0.613 | Low |
| Q9UNE7 | STUB1 | Q9UPY5 | 1.00 | 2.88 | 1.00 | 2.12 | 0.613 | Low |
| Q8TBB1 | LNX1 | Q9UPY5 | 1.00 | 2.88 | 1.00 | 2.12 | 0.613 | Low |
| Q86Y01 | DTX1 | Q9UPY5 | 1.00 | 2.88 | 1.00 | 2.12 | 0.613 | Low |
| P62873 | GNB1 | Q9UPY5 | 1.00 | 2.88 | 1.00 | 2.12 | 0.613 | Low |

## Supplementary Figures

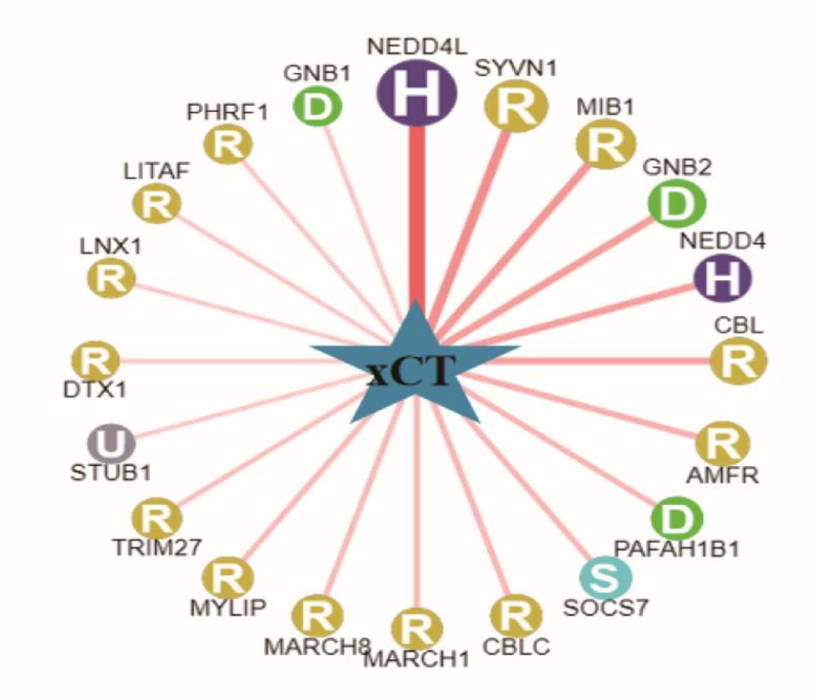


**Figure S1. UbiBrowser database showed NEDD4L is the highest confidence to interact with xCT.**

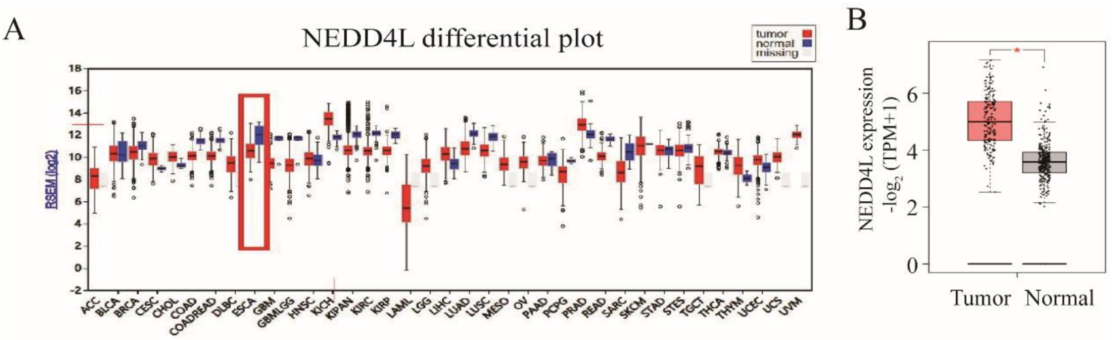


**Figure S2. NEDD4L expression in public database. A** Analysis of TCGA database showed that NEDD4L mRNA levels were significantly decreased in esophageal cancer compared with normal esophageal tissue. **B** GEPIA database results indicated that NEDD4L mRNA levels were increased in esophageal cancer (n = 182) compared to normal tissue (n = 286).

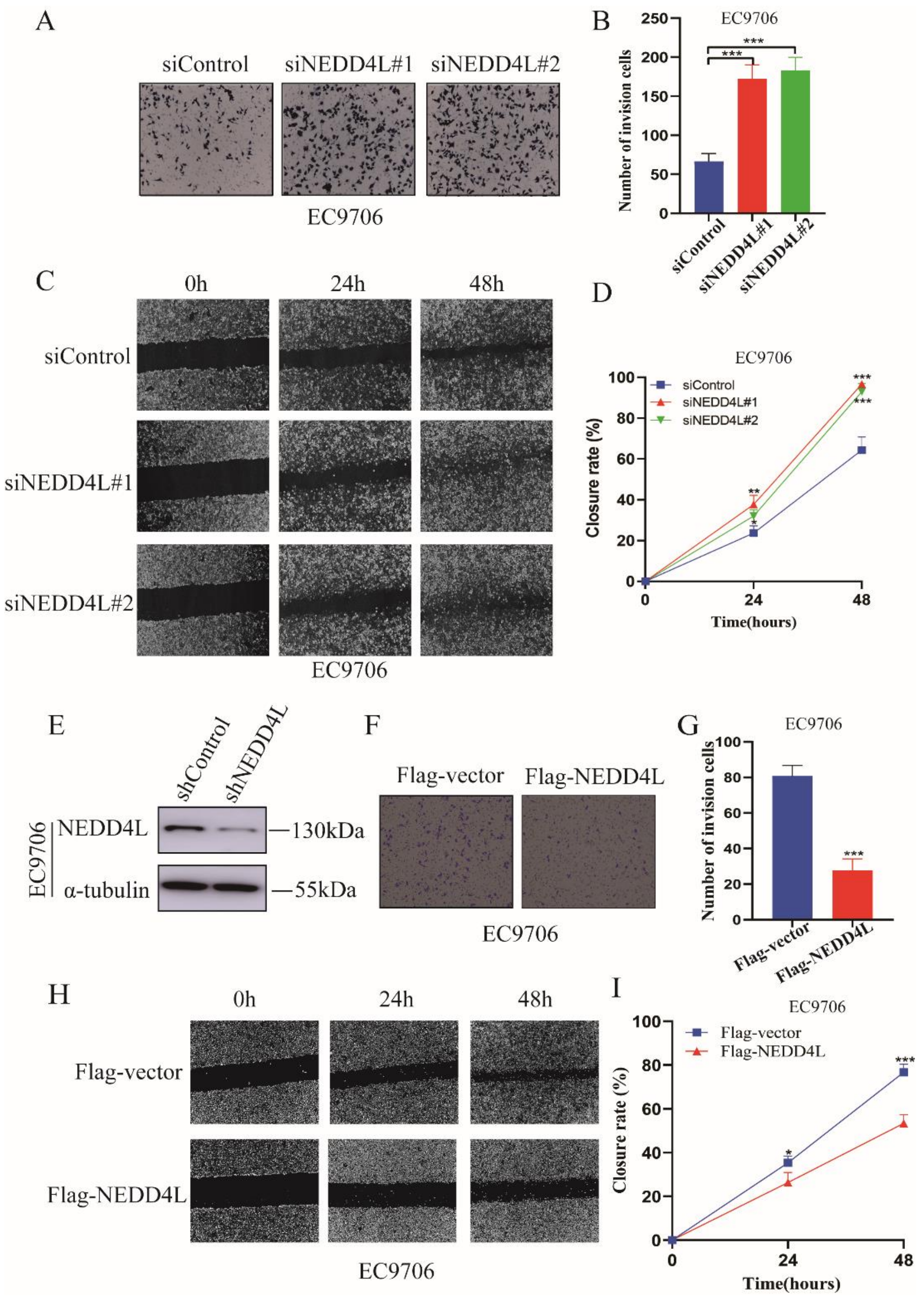


**Figure S3. The impact of silencing NEDD4L and overexpressing NEDD4L on invasion capacity and wound closure speed in ESCC cells.** **A, B** silencing NEDD4L promoted cell invasion in EC9706 cells. n = 3 per group. **C, D** silencing NEDD4L promoted wound closure speed in EC9706 cells. n = 3 per group**. E** shNEDD4L transfection efficiency in EC9706 cell line. **F, G** Overexpression NEDD4L suppressed cell invasion in EC9706 cells. n = 3 per group. **H, I** Overexpression

NEDD4L inhibited wound closure speed in EC9706 cells. n = 3 per group. The data are presented as the mean ± SD. Statistical significance was determined by one-way ANOVA. *$p < 0.05$; **$p < 0.01$; ***$p < 0.001$.

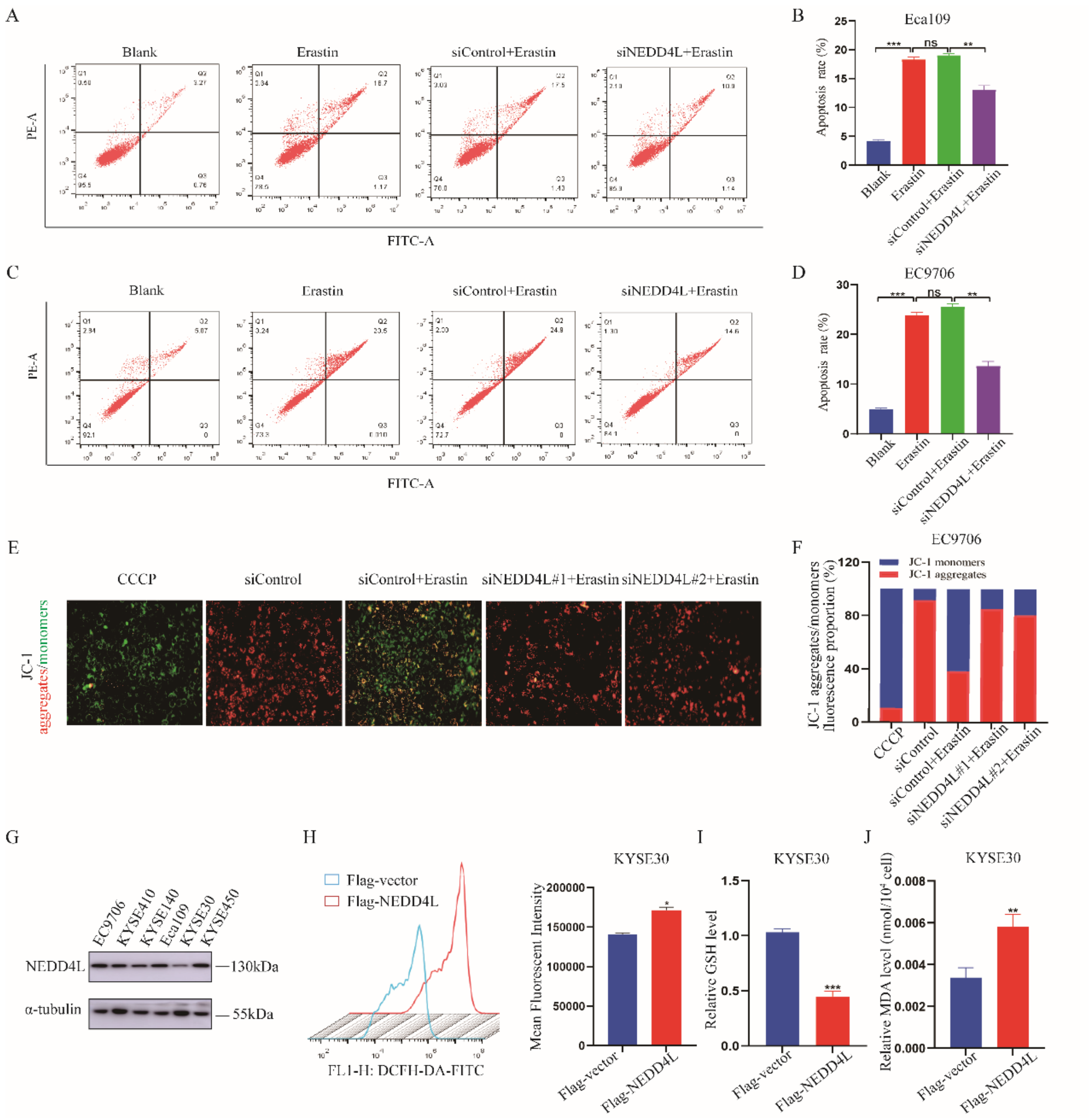


**Figure S4. The influence of silencing NEDD4L and overexpressing NEDD4L on ferroptosis. A-D** Cell death rate after treated with erastin for 24 h and transfected with siControl/siNEDD4L for 48 h were detected in Eca109 and EC9706 cells. n = 3 per group. **E, F** The effect of silencing NEDD4L on MMP under the treatment of erastin in

EC9706 cells was detected by optical microscopy. **G** NEDD4L expression in EC9706, Eca109, KYSE30, KYSE140, KYSE410 and KYSE450 cells was measured by western blotting. **H** ROS level in KYSE30 cells transfected with Flag-vector or Flag-NEDD4L plasmids for 48 h was detected. n = 3 per group. **I** GSH level in KYSE30 cells transfected with Flag-vector or Flag-NEDD4L plasmids for 48 h was detected. n = 3 per group. **J** MDA content in KYSE30 cells transfected with Flag-vector or Flag-NEDD4L plasmids for 48 h was detected. n = 3 per group. The data are presented as the mean ± SD. Statistical significance in **B, D, F** were determined by one-way ANOVA. *$p < 0.05$; **$p < 0.01$; ***$p < 0.001$. Statistical significance in **H-J** Statistical analysis were performed using Student's t-test. *$p < 0.05$; **$p < 0.01$; ***$p < 0.001$; ns, not significant.

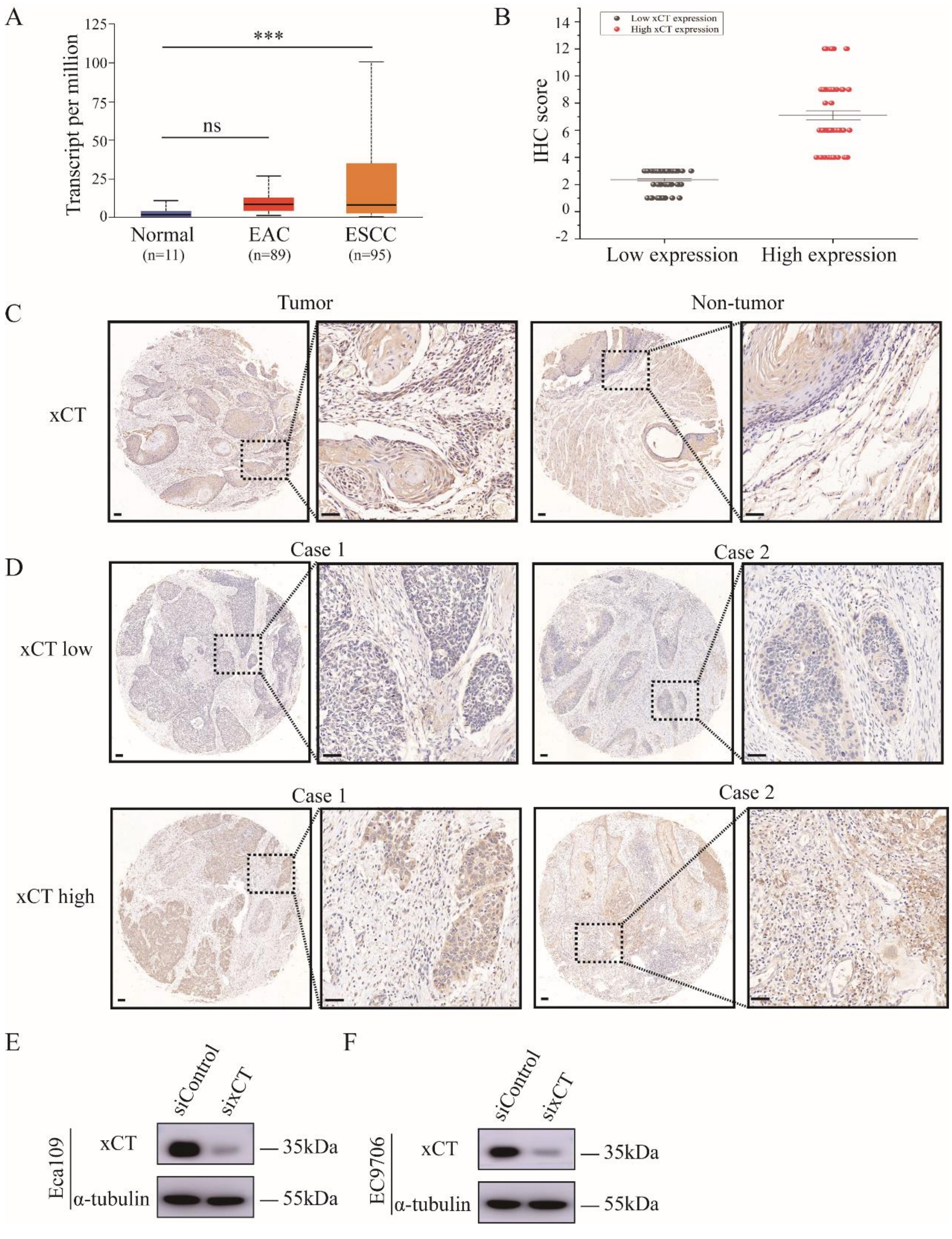


**Figure S5. The expression and IHC score of xCT in 142 ESCC cases.**

**A** UALCAN database results indicated that xCT mRNA levels is significantly increased in ESCC (n = 95) compared to normal tissue (n = 11). **B** xCT expression score in 142 cases of ESCC (n = 142). **C** xCT protein expression was increased in ESCC tissues (n = 25) compared with normal tissue (n = 25) as analyzed by IHC. Scale bars, 100 μm. **D**

Representative images of xCT low expression and high expression analyzed by IHC (n = 142). Scale bars, 100 μm. **E, F** xCT silencing efficiency in Eca109 and EC9706 cells were evaluated by western blotting.

## Supplementary materials and methods

### Wound healing assay

EC9706 cells were transfected with 50 nM NEDD4L siRNA or 5 μg Flag-NEDD4L plasmids for 24 h. Then, cells were transfer into 24-well plates and cultured with 1% FBS. 10 μ L pipette tip was used for scratching when the cell density reached 100%. Wound distance was measured at 0 hour, 24 hours and 48 hours and normalized with the distance at 0 hour. Image J software was used to analyse the recovery speed.

### Invasion assay

EC9706 cells were transfected with 50 nM NEDD4L siRNA or 5μg NEDD4L plasmids. To induce invasion, Matrigel (356234, BD) was applied to the upper chamber. Following a 12-hour incubation period, the cells were delicately extracted. After that, cells were fixed by 4% paraformaldehyde and stained by crystal violet. Image J software was used for counting the number of invasive cells.

### Cell death assay

Eca109 and EC9706 cells transfected with siNEDD4L/siControl were treated with erastin (20 μM) for 24 h. Then, cells were collected and re-suspended with staining buffer containing 5 μL Annexin V, FITC and 5 μL PI solution (AD10, DOJINDO). Afterwards, the cells incubated at room temperature in dark for 15 minutes, followed by detection using flow cytometry (Beckman, America).

**Mitochondrial membrane potential (MMP) assay**

JC-1 kit (C2006, Beyotime) was used to detect MMP. EC9706 cells were transfected with 50 nM siNEDD4L or siControl for 24 h. Subsequently, erastin with final concentration of 20 μM was added into the wells. After 24 h treatment, cells were gently washed with PBS. 1mL of fresh culture medium with JC-1 dyeing working solution (25 μM) were added and mixed well. In addition, cells were treated with carbonyl cyanide-m-chloro phenylhydrazone (CCCP) at a concentration of 10 μM for 20 minutes as a positive control. Afterwards, the cells were incubated in the cell incubator at 37℃ in a dark environment for 20 min, followed by being washed twice with 1× JC-1 staining buffer. Then, 2 mL fresh culture medium was added, and fluorescence intensity was observed by a fluorescence microscope.

# Original western blot

Fig. 2A

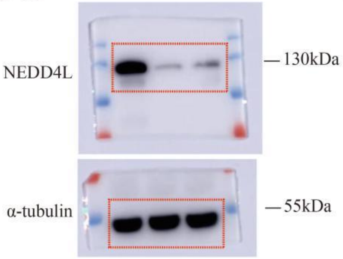


Fig. 2B

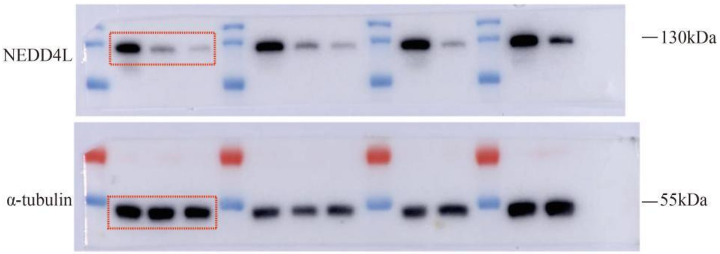


Fig. 2J

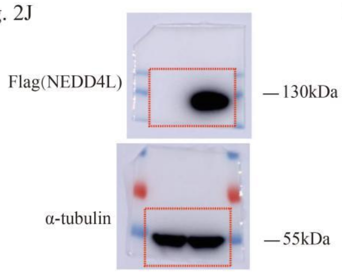


Fig. 2K

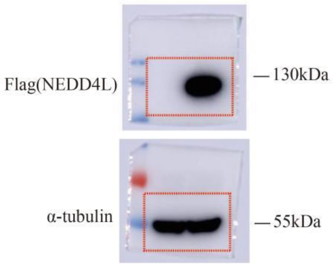


Fig. 4D

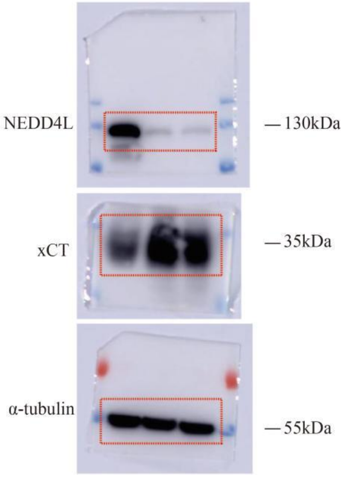


Fig. 4E

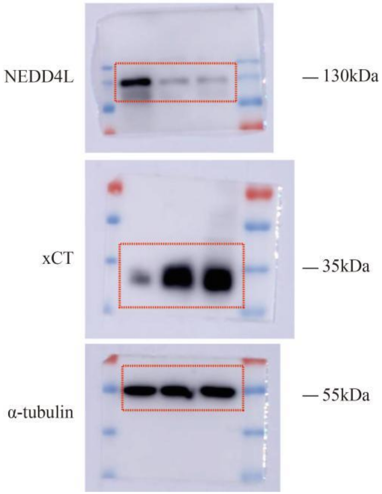

Fig. 4F

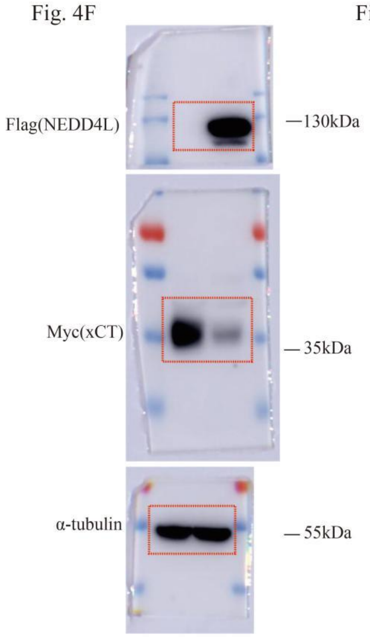


Fig. 4G

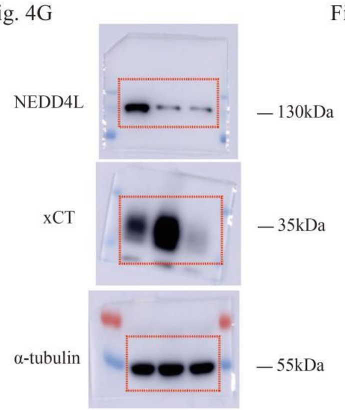


Fig. 4H

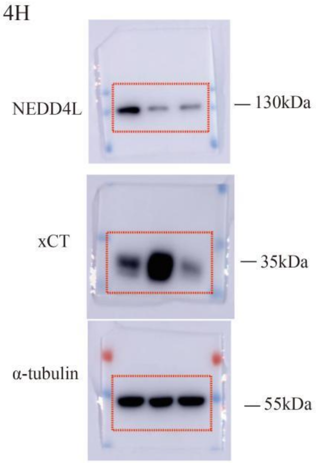


Fig. 5B

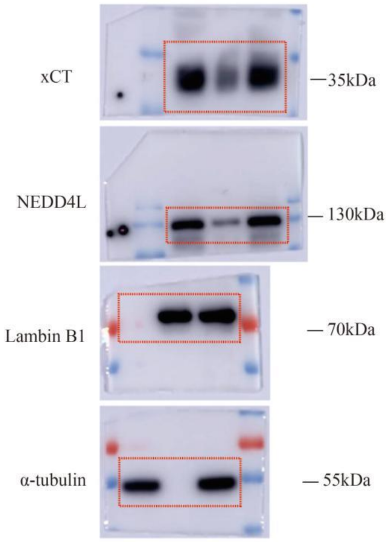


Fig. 5C

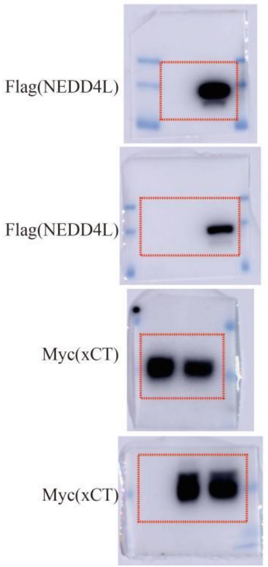


Fig. 5D

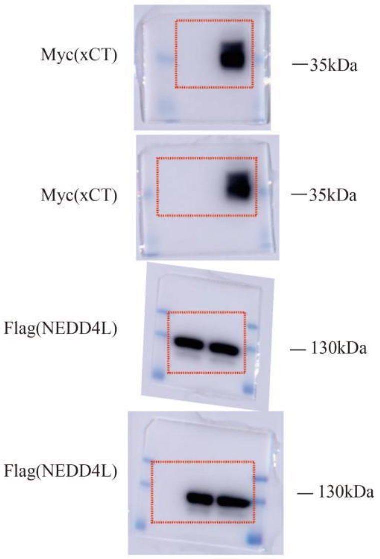

Fig. 5G

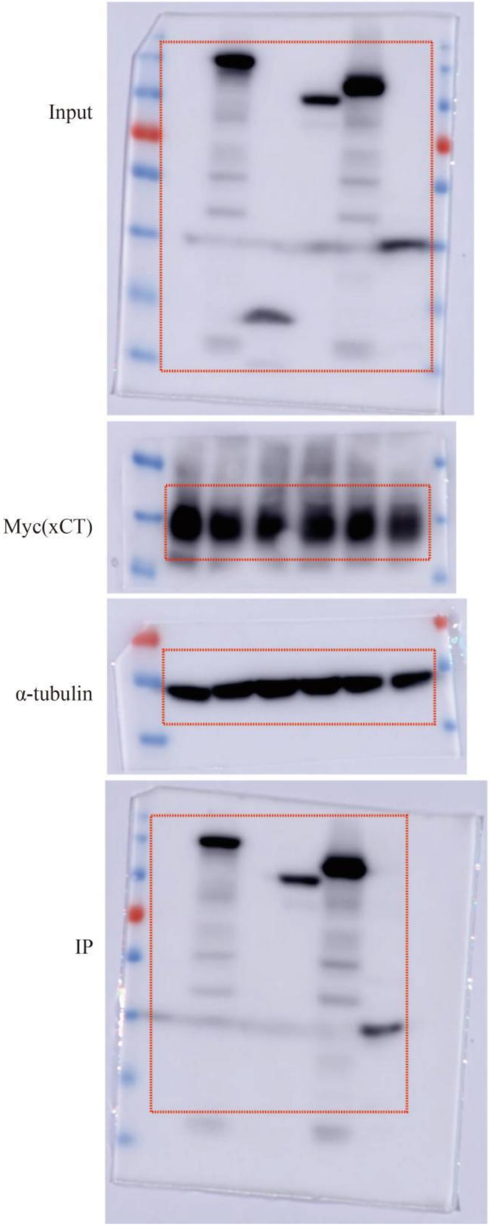


Fig. 5H

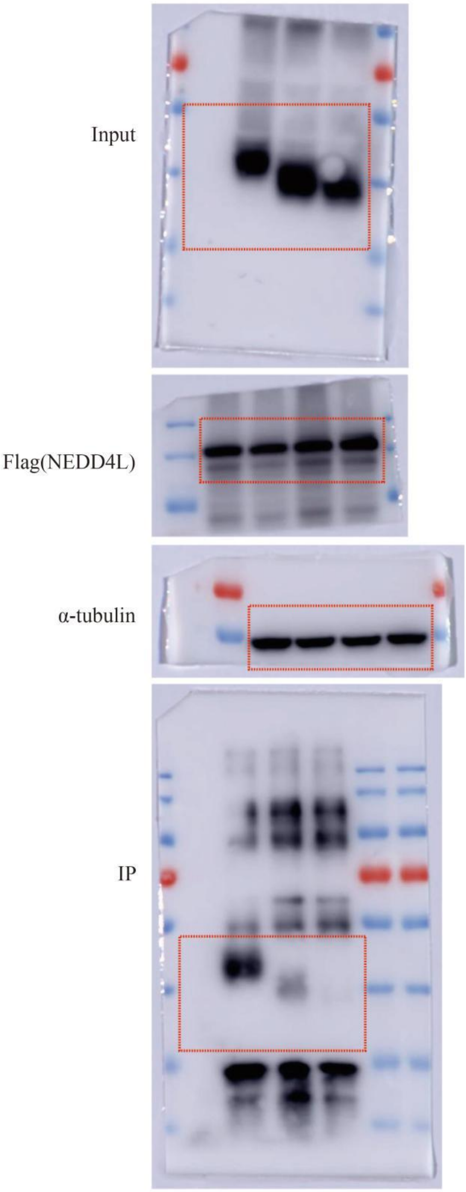

Fig. 6A

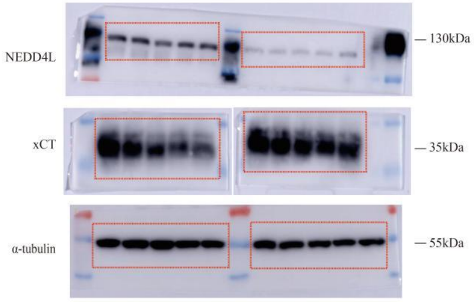


Fig. 6C

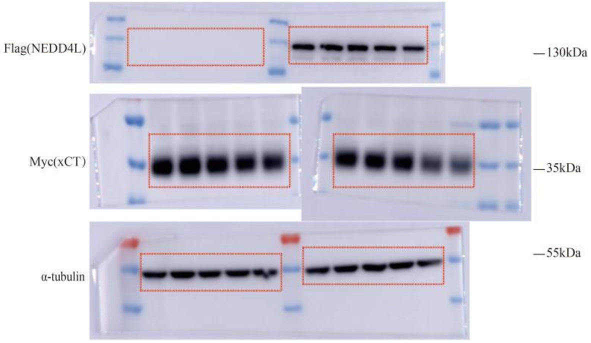


Fig. 6E

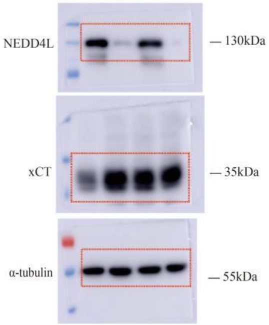


Fig. 6F

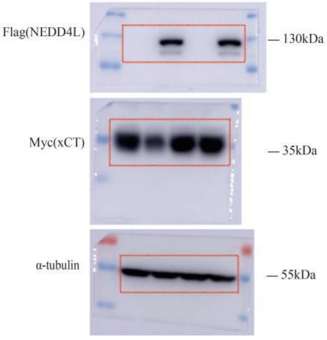

Fig. 6G

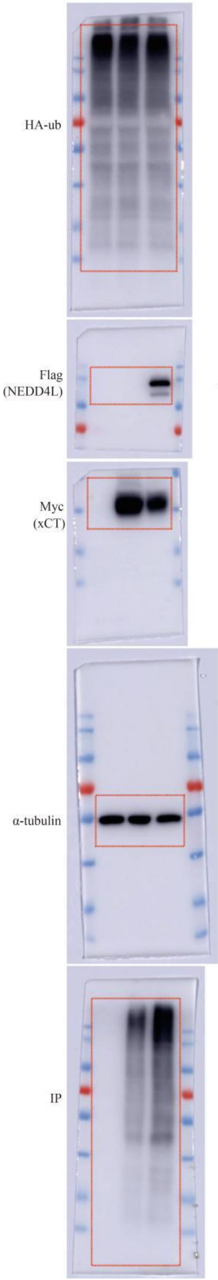


Fig. 6H

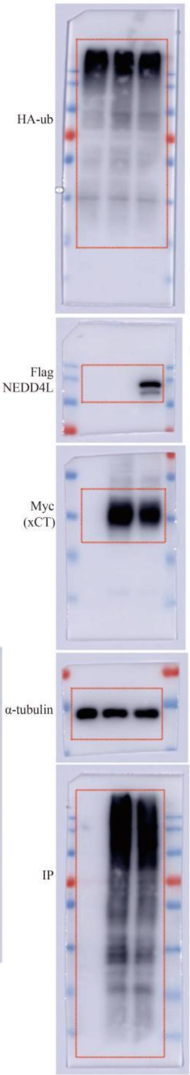


Fig. 6I

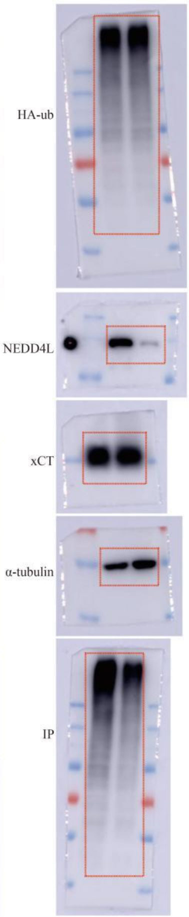


Fig. 6J

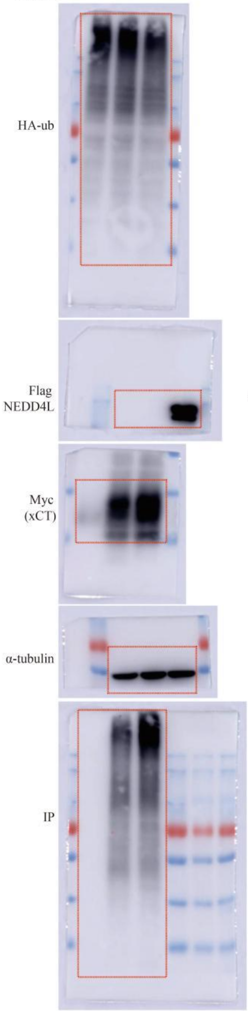


Fig. 6K

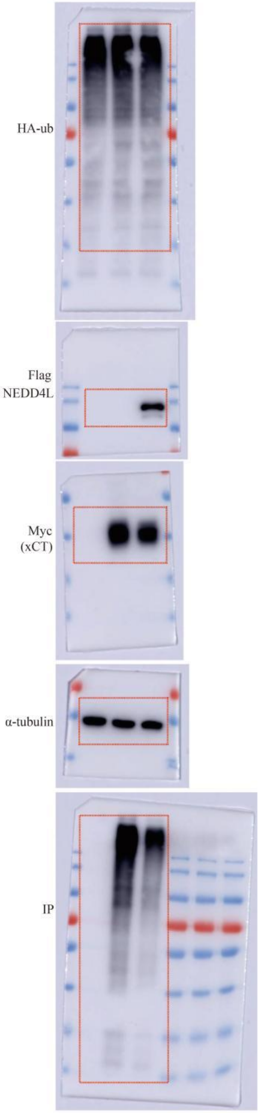

Fig. 6L

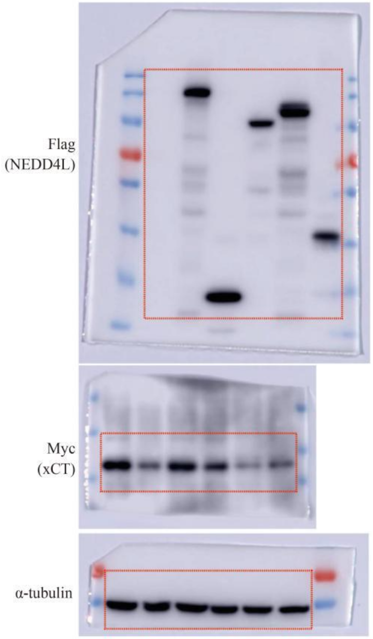


Fig. 6M

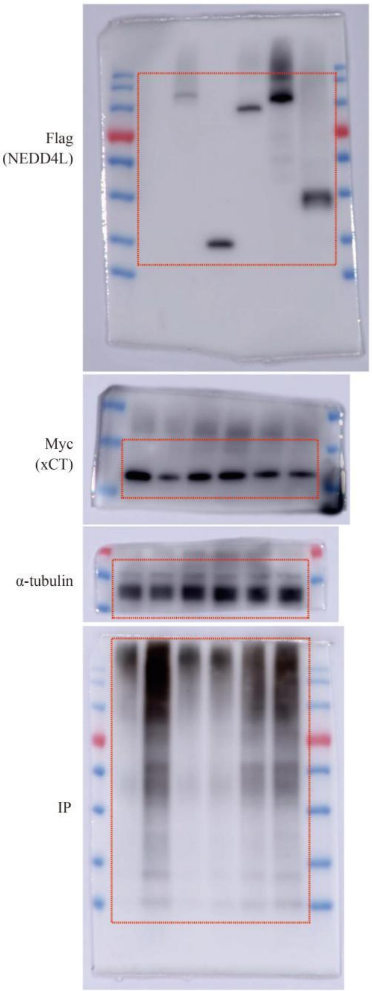


Fig. 6Q

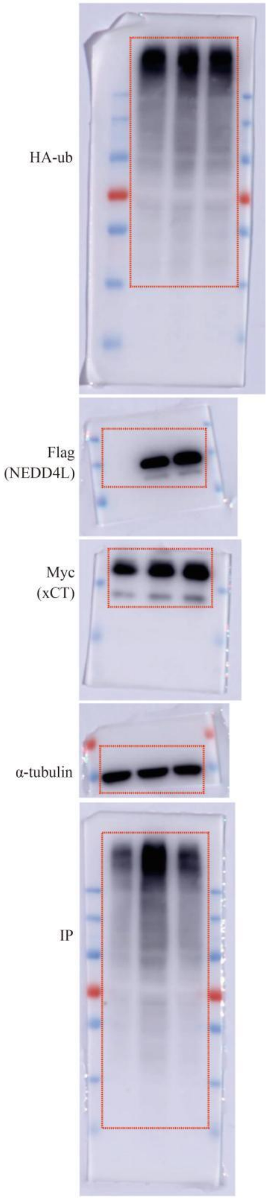


Fig. 6N

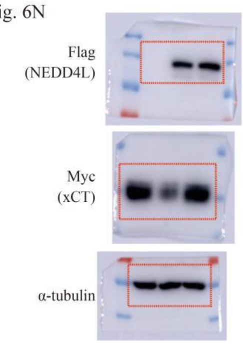


Fig. 6O

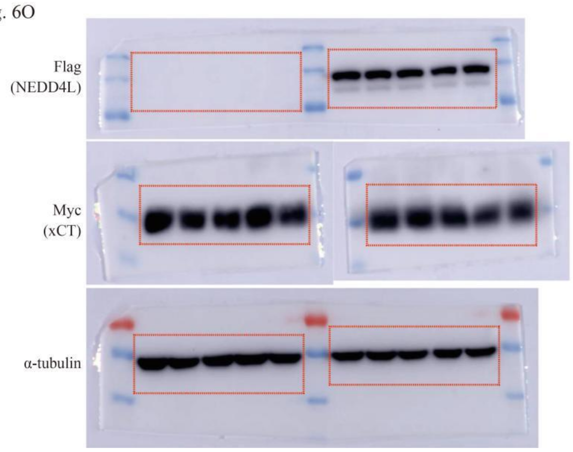

## Fig. S3E

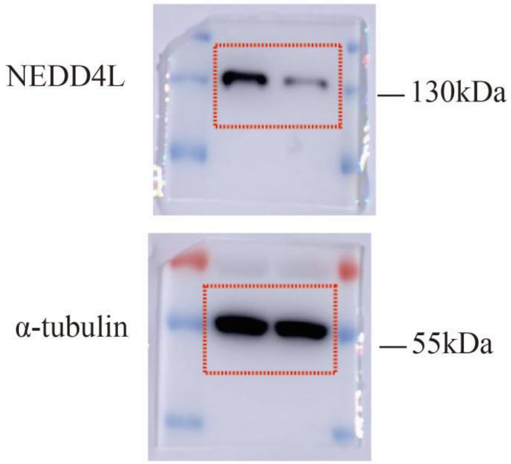


## Fig. S4G

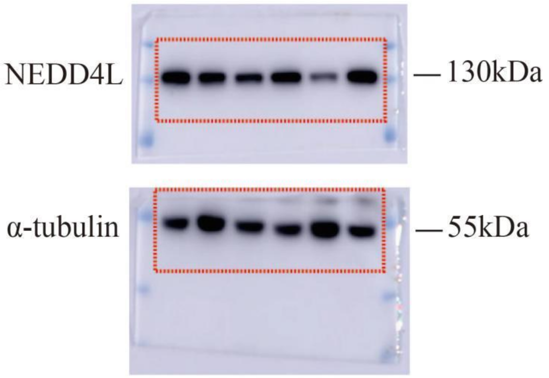


## Fig. S5E

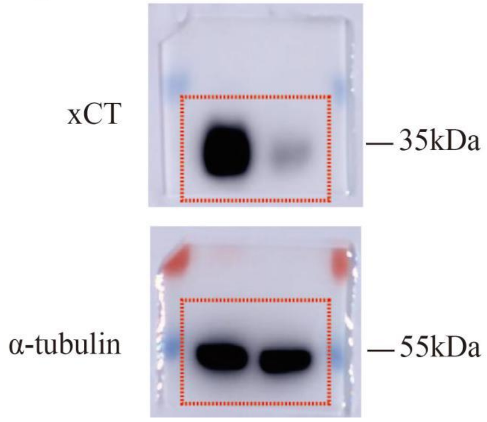


## Fig. S5F

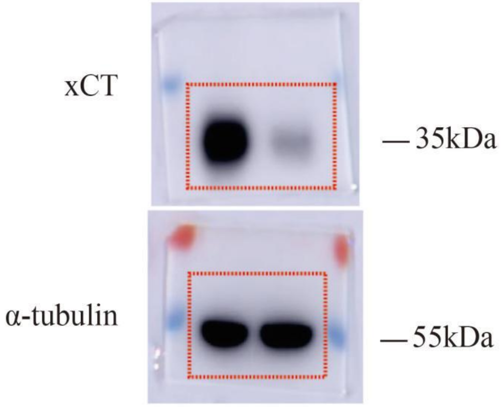

Supplement: Supplementary file 1 — Supplementary materials [file 41420_2024_2243_MOESM1_ESM.pdf]
